# A systematic review and meta-analysis on prevalence and distribution of *Taenia* and *Echinococcus* infections in Ethiopia

**DOI:** 10.1186/s13071-021-04925-w

**Published:** 2021-09-06

**Authors:** Nigus Abebe Shumuye, John Asekhaen Ohiolei, Mebrahtu Berhe Gebremedhin, Hong-Bin Yan, Li Li, Wen-Hui Li, Nian-Zhang Zhang, Bao-Quan Fu, Wan-Zhong Jia

**Affiliations:** 1 State Key Laboratory of Veterinary Etiological Biology/National Animal Echinococcosis Para-Reference Laboratory/Key Laboratory of Veterinary Parasitology of Gansu Province/Lanzhou Veterinary Research Institute, CAAS, Lanzhou, 730046 People’s Republic of China; 2 Department of Veterinary Clinical Medicine and Epidemiology, College of Veterinary Sciences, Mekelle University, Kalamino campus, P.O. Box 2084, Mekelle, Tigray Ethiopia; 3 Key Laboratory of Animal Parasitology of Ministry of Agriculture, Shanghai Veterinary Research Institute, CAAS, Shanghai, 200241 People’s Republic of China; 4 Jiangsu Co-Innovation Center for Prevention and Control of Important Animal Infectious Disease, Yangzhou, 225009 People’s Republic of China

**Keywords:** Cystic echinococcosis, Taeniasis, Cysticercosis, Epidemiology, Risk factors, Ethiopia

## Abstract

**Background:**

Tapeworm infections are among the tropical neglected parasitic diseases endemically occurring in Ethiopia. This systematic review and meta-analysis aims at estimating the pooled prevalence and distribution of *Taenia* and *Echinococcus* infections in humans and animals from reports from Ethiopia.

**Methods:**

The systematic search was conducted in four bibliographic databases (PubMed, Google Scholar, Africa Journal Online and Science Direct). Additional data were retrieved from grey literature. Studies that met the inclusion criteria were considered for the systematic review and meta-analysis. The meta-analysis was conducted using MetaXL add-in for Microsoft Excel. Heterogeneity and inconsistency were evaluated using Cochran’s *Q* and *I*^2^ statistics, respectively.

**Results:**

The study provides a country-based database of *Taenia* and *Echinococcus* infections consisting of 311 datasets from 201 publications which were mostly abattoir surveys; of these, 251 datasets were subjected to meta-analysis. Most of the studies were from Oromia (32.8%) followed by Amhara (22.9%) regional states. The pooled prevalence of cystic echinococcosis in intermediate and accidental hosts was calculated as 22% (95% CI 18–26%) and high study variability (*Q* = 24,420.65, *I*^2^ = 100%, *P* = 0.000). Moreover, a pooled prevalence of *Echinococcus* infections in final hosts was calculated as 33% (95% CI 20–48%) and low study variability (*Q* = 17.24, *I*^2^ = 65%, *P* = 0.001). Similarly, study subjects (human, cattle, sheep, goat and wolf) were infected by *Taenia* spp. with pooled prevalence of 3% (95% CI 2–4%) and moderate study variability (*Q* = 279.07, *I*^2^ = 89, *P* = 0.000). Meanwhile, the pooled prevalence of *Taenia hydatigena*, *T. ovis* and *T. multiceps* infections in intermediate hosts were calculated as 38%, 14% and 5%, respectively. The random effect meta-analysis of bovine cysticercosis showed a pooled prevalence of 7% (95% CI 5–9%) and high study variability was of (*Q* = 4458.76; *I*^2^ = 99%, *P* = 0.000). Significant differences in prevalence of *Taenia* and *Echinococcus* infections between study sites or different livestock origins have been reported.

**Conclusion:**

The study evidenced a comprehensive dataset on the prevalence and distribution of *Taenia* and *Echinococcus* infections at different interfaces by regions and hosts and hence can aid in the design of more effective control strategies.

**Graphical Abstract:**

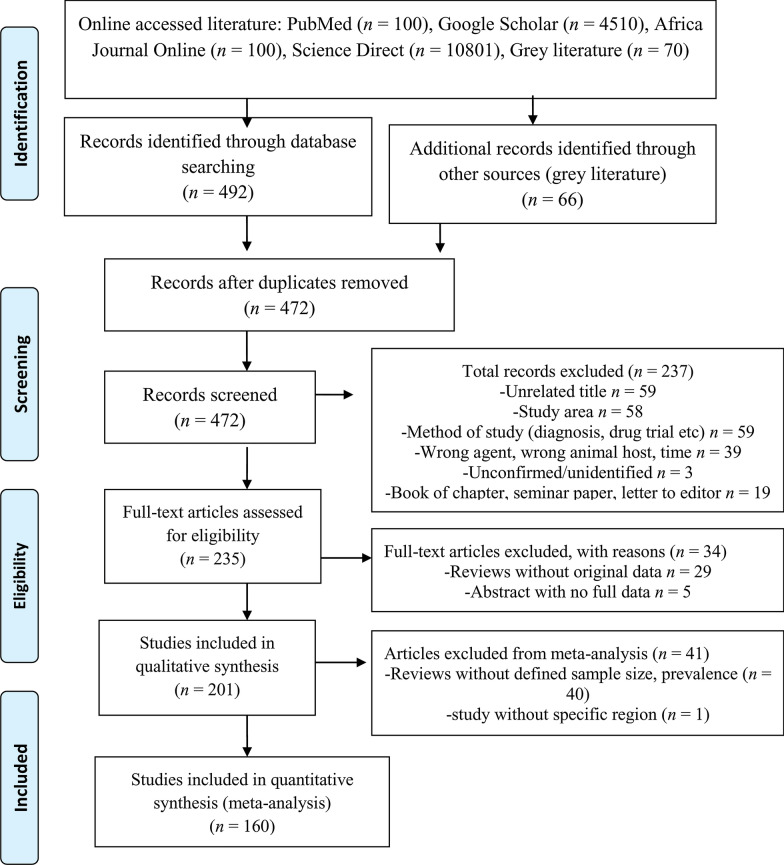

**Supplementary Information:**

The online version contains supplementary material available at 10.1186/s13071-021-04925-w.

## Background

Parasitic diseases are highly prevalent in resource-poor Sub-Saharan African (SSA) countries and not only cause severe economic losses but also adversely affect public health [1]. Ethiopia is a SSA country with a population of 112,078,730 [2]. It is also host to about 60.39 million cattle, 31.3 million sheep, 32.74 million goats, 2.01 million horses, 8.85 million donkeys, 0.46 million mules and about 1.42 million camels [3]. The livestock production system in the country is characterized by mixed agriculture (animal and crop production) where there is high livestock-dog contact that can enhance the spread of parasites [4]. In Ethiopia about 80% of households have direct contact with domestic animals, creating an opportunity for zoonotic disease transmission [5–7]. Tapeworm infections are among the tropical neglected parasitic diseases endemic in Ethiopia [8, 9].

Tapeworms (or cestodes) characterized by body differentiation with scolex, neck and strobili are an important group of flatworms that parasitize humans, livestock and other susceptible animals [10, 11]. *Taenia* and *Echinococcus* species are tapeworms classified under phylum Platyhelminthes, subclass Eucestoda, order Cyclophyllidea and family Taeniidae. Regarding the general morphology of these parasites, the scolex possesses a rostellum usually armed with double rows of hooks and unpaired genitalia in each proglottid with irregularly alternating marginal genital pores. The eggs have a radially striated hardened ‘shell’ (embryophore) [12]. They have an indirect life cycle where adults occupy the small intestine of carnivores and humans while the larva stages occur in various organs of different mammals (including humans) that serve as intermediate hosts. The complex life cycle of tapeworms starts with a larval stage and usually involves several hosts [13]. The metacestode of *Echinococcus* shows a low degree of host specificity and has a much greater reproductive potential compared to the *Taenia* species [12].

*Taenia solium*, *T. saginata* and *T. asiatica* are cestodes that cause taeniasis in humans and cysticercosis in intermediate host animals (cows and pigs) [14]. Beef is a source of *T. saginata* infection, while pork and pig viscera are responsible for *T. solium* and *T. asiatica* infections [15]. *T. asiatica* is a sister species of *T. saginata* [16] that is commonly found in Asian countries [17, 18]. *Taenia hydatigena* (*Cysticercus tenuicollis*) and *T. ovis* (*C. ovis*) are species occurring mainly in small ruminants [19, 20]. The larval stage of *Taenia multiceps* (*Coenurus/Cysticercus cerebralis*) causes coenurosis (gid or sturdy) in small ruminants. The larvae develop in the brain and spinal cord of sheep, goats and sometimes cattle. The larval development in the brain has also been reported in humans and horses with cerebral manifestation [21, 22]. In general, the transmission of many important cestodes in livestock, such as *Taenia* spp. and *Echinococcus* spp., usually involves ‘predator-prey’ relationships between carnivores (final hosts) or omnivores and herbivores (intermediate hosts) and humans as accidental hosts in the case of echinococcosis [23].

Cystic echinococcosis (CE), the most common form of echinococcosis in human and domesticated animals, is caused by *E. granulosus * sensu lato. It is the least severe and most treatable form of echinococcosis since the larvae usually develop as isolated single cysts. In contrast, alveolar echinococcosis (AE), caused by *E. multilocularis*, is less common but more fatal and difficult to treat. The larvae of this organism grow as multiple budding cysts, and the involvement of wildlife in the lifecycle makes it difficult to prevent [24–26].

Metacestodes of both *Taenia* and *Echinococcus* are responsible for downgrading and lowering the quantity and quality of animal commodities [4, 19]. The economic burden of CE on the global livestock industry alone has been estimated to be > $2 billion per annum due to the condemnation of edible carcasses and offal such as liver, lung and heart [27]. In Ethiopia, significant degrees of financial losses were estimated at various levels in different locations. For example, reports estimate annual losses ranging from $2807.89 in Tigray [28] to $131,737.19 in Hawassa, South Nation and Nationality of People (SNNP) [29] based on abattoir surveys due to CE and bovine cysticercosis. Furthermore, average annual losses of 4,937,583.21 Ethiopian birr (ETB) or $225,036.97 due to taenicidal drugs for human treatment were estimated in Ethiopia [30].

Globally, echinococcosis presents a serious health concern especially in endemic countries with increasing infection rate resulting from an increase in the population of definitive hosts [31, 32]. The long-standing tradition of eating raw meat (beef) in Ethiopia has led to a craving for raw beef in most of the people. The close relationship among dogs, sheep and humans maintains the infection by completing the parasite's life cycle. Absence of rigorous meat inspection procedures, predominant home slaughtering of animals, the habit of feeding domesticated dogs with condemned offal and the subsequent contamination of pasture and grazing fields facilitate the maintenance of the life cycle and play an important role in the transmission of these zoonotic parasites.

Although several reports are available on various aspects of taeniasis and echinococcosis in Ethiopia, data on prevalence and distribution are affected by the type of study population, sample size, study design and other epidemiological factors such as host, pathogen and/or environmental factors. Thus, it is important to attain a comprehensive and larger scale overview and identify possible forecasters of the parasitic infection dynamics in the population of interest to provide national strategies for the control of taeniasis and echinococcosis. This systematic review and meta-analysis aims at estimating the pooled prevalence and distribution of taeniasis and echinococcosis in human and animal from reports from different parts of the country.

## Materials and methods

### Study area

Ethiopia is a rugged and landlocked country in the Horn of Africa, crossed by the Great Rift Valley, and borders Eritrea to the north, Djibouti and Somalia to the east, Sudan and South Sudan to the west, and Kenya to the south. The country covers an area of 1,126,829 km^2^ and is located between 9.1450°N and 40.4897°E. There are nine regions and two chartered cities [33]. In Africa, Ethiopia is the second most populated country after Nigeria and is known for its huge livestock population [2, 3].

### Study protocol

A protocol addressing the review questions was developed by defining outcomes of interest and inclusion/exclusion criteria. Studies related to the occurrence, incidence and prevalence of taeniasis (due to *Taenia solium*, *T. saginata*, *T. hydatigina*, *T. ovis* and *T. multiceps*) and echinococcosis in humans and domesticated animals were analysed.

Inclusion criteria including manuscripts written in English which were either published or grey literature, time/period (1990–2020), geographical location (Ethiopia), study subject (human and/or domesticated animal such as cattle, sheep, goat, pig, camel and dog) and design (cross-sectional study, case report and short communication), dissertations and theses were also included.

Exclusion criteria included unrelated data, duplicated, wrong pathogen/agent of interest, case control study design, experimental study (development of diagnostics, drug efficacy, ethnobotanical study), KAP (knowledge, attitude and practice) questionnaire based study, book chapters, absence of original data, books, review articles without original data, editorials or letters to the editor without original data, all data before 1990, and unavailable full text or abstract only papers.

Article selection was carried out using a three-step process: first, duplicate articles were removed, then titles and/or abstracts were screened for relevance to the topic, and finally full texts were screened for eligibility. The quality standard of each manuscript was assessed independently by two authors. Disagreements or uncertainties were resolved through discussion with other reviewers. The study approach ensured compliance with methodological recommendations of the preferred reporting items for systematic reviews and meta-analysis (PRISMA) guideline for review processes [34] and a PRISMA check list is provided as Additional file 1: Table S1.

### Searching strategy

Searching was done systematically in four bibliographic databases including PubMed, Google Scholar, Africa Journal Online and Science Direct with search terms and key elements using Boolean operators: tapeworm OR taenia/taeniasis/taeniosis/cysticercosis/coenurosis* OR echinococc/hydatid cyst/hydatidosis* AND Ethiopia. The keywords/strings were rearranged to phrases close to outcome of interest. To increase the chance of recovering additional data, articles were retrieved from the reference section and citation lists of the full texts such as original research articles and reviews. Different combinations were tailored for each electronic database to narrow the amount of results retrieved but at the same time maximizing the number of relevant studies. The last search was conducted on June 30, 2020.

### Data extraction

Data were extracted by two independent researchers and any disagreements were resolved by consensus among the researchers using the standardized extraction forms to guarantee consistency and accuracy. Data were extracted from eligible studies using standardized Microsoft Excel tables (Microsoft Office 2010). The data extracted included paper identification, brief study description (study area, year of study, species, sex, age), study design, diagnostic method (parasitological, ultrasound, surgery, molecular identification and serology), total sample size, number of infected/positive, prevalence and respective 95% confidence interval, and parasitic category. All patient medical data analysed in this study were anonymised and extracted from publications in which they were reported in an aggregated form as case or population counts. Approval from an ethical committee or institutional review board was not necessary for this study.

### Statistical analysis

The extracted data on Excel sheets were analysed qualitatively and quantitatively. The prevalence data were analysed using the total sample size and number of positives. Studies with known sample size and number of positive samples were minimum requirements for further meta-analysis. However, data sources from retrospective studies were not included in the meta-analysis because such data might not indicate the true nature (exact value) of the infections in the study area.

The meta-analysis procedure was performed for each potential risk factor [host, pathogen, environment (region)] and visualised using forest plots as described by [35]. Briefly, we estimated the pooled prevalences with 95% confidence intervals (CI) for *Taenia* and *Echinococcus* infections in intermediate and final hosts, which were further analysed by regions. The meta-analysis was conducted using MetaXL add-in for Microsoft Excel (EpiGear International, Queensland, Australia), and results were presented as a forest plot diagram, which shows estimates of pooled prevalence and their respective CIs of individual studies with the summary measure. The results of the analyses are presented with their *P*-values. The threshold for statistical significance was set at *P* < 0.05. It was calculated using the random-effects model, which uses the inverse of the sampling variance and a constant variable across the population effects to weight each study [36, 37]. Cochran’s *Q* and *I*^2^ statistics were used for evaluation of heterogeneity and inconsistency, respectively. If the *P*-value of the *Q* test was < 0.05 and *I*^2^ was > 50%, heterogeneity was inferred [38]. Finally, publication bias was assessed by the Luis Furuya-Kanamori (LFK) index and funnel plot [39]. An LFK index within the range of ± 1, ± 2 and outside ± 2 was inferred as symmetrical, slightly/minor asymmetrical and significantly/major asymmetrical, respectively, where symmetrical index indicates the absence of publication bias [40].

## Results

### Qualitative analysis

**Fig. 1:**
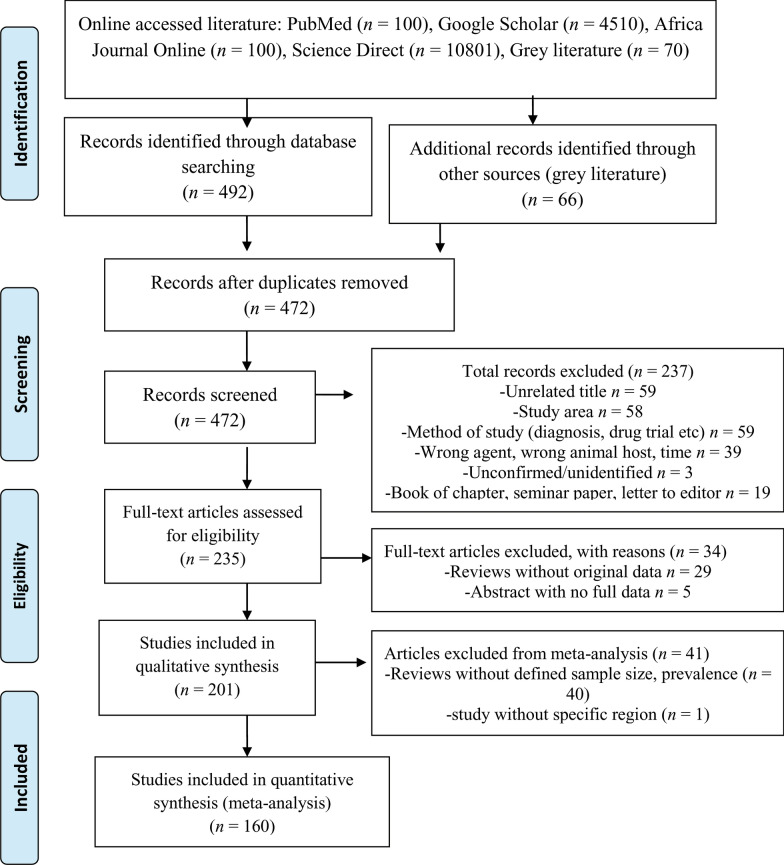
Study flowchart for the prevalence and distribution of *Taenia* and *Echinococcus* infections in Ethiopia

The online literature search yielded 15,581 potentially relevant references. After the first screening by title and/or abstract, the full texts of 558 remaining publications were further examined. A total of 86 duplicate articles were excluded while a further 271 articles were excluded during the second selection process. Unrelated title, study area and purpose/method of study were the three most common reasons for exclusion. A total of 201 publications resulting in 311 datasets were found eligible for inclusion in this systematic review, of which 251 datasets were subjected to meta-analysis to determine associations between *Taenia* and/or *Echinococcus* infection and potential risks. The review process for selecting the articles is shown in the flow diagram (Fig. 1).

**Fig. 2:**
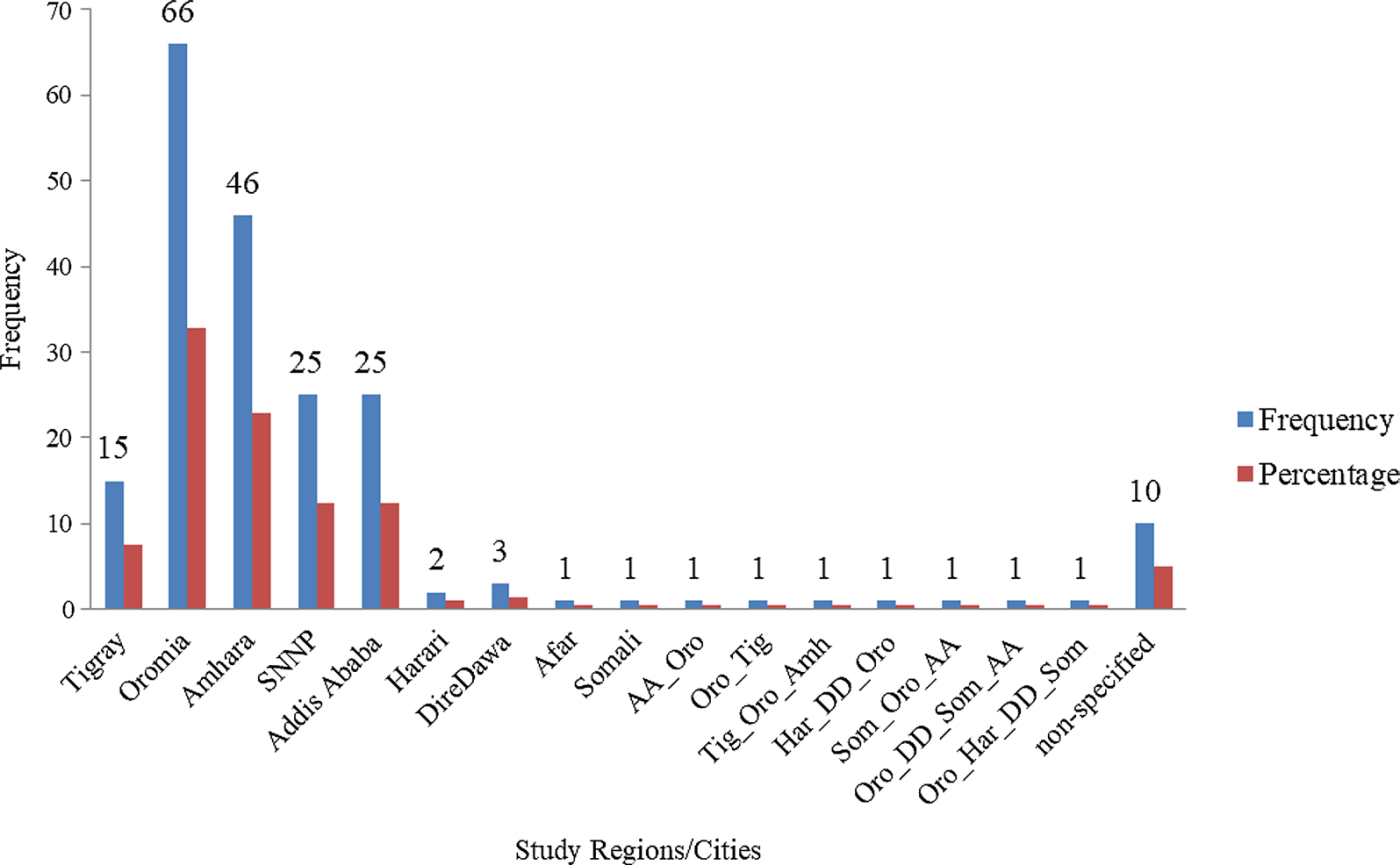
Overall distributions of reports for the prevalence and distribution of *Taenia* and *Echinococcus* infections in Ethiopia. *AA* Addis Ababa, *Oro* Oromia, *Tig* Tigray, *SNNP* Southern Nation and Nationality of People, *Amh* Amhara, *Har* Harar, *DD*, Dire Dawa, *Som* Somali

**Fig. 3:**
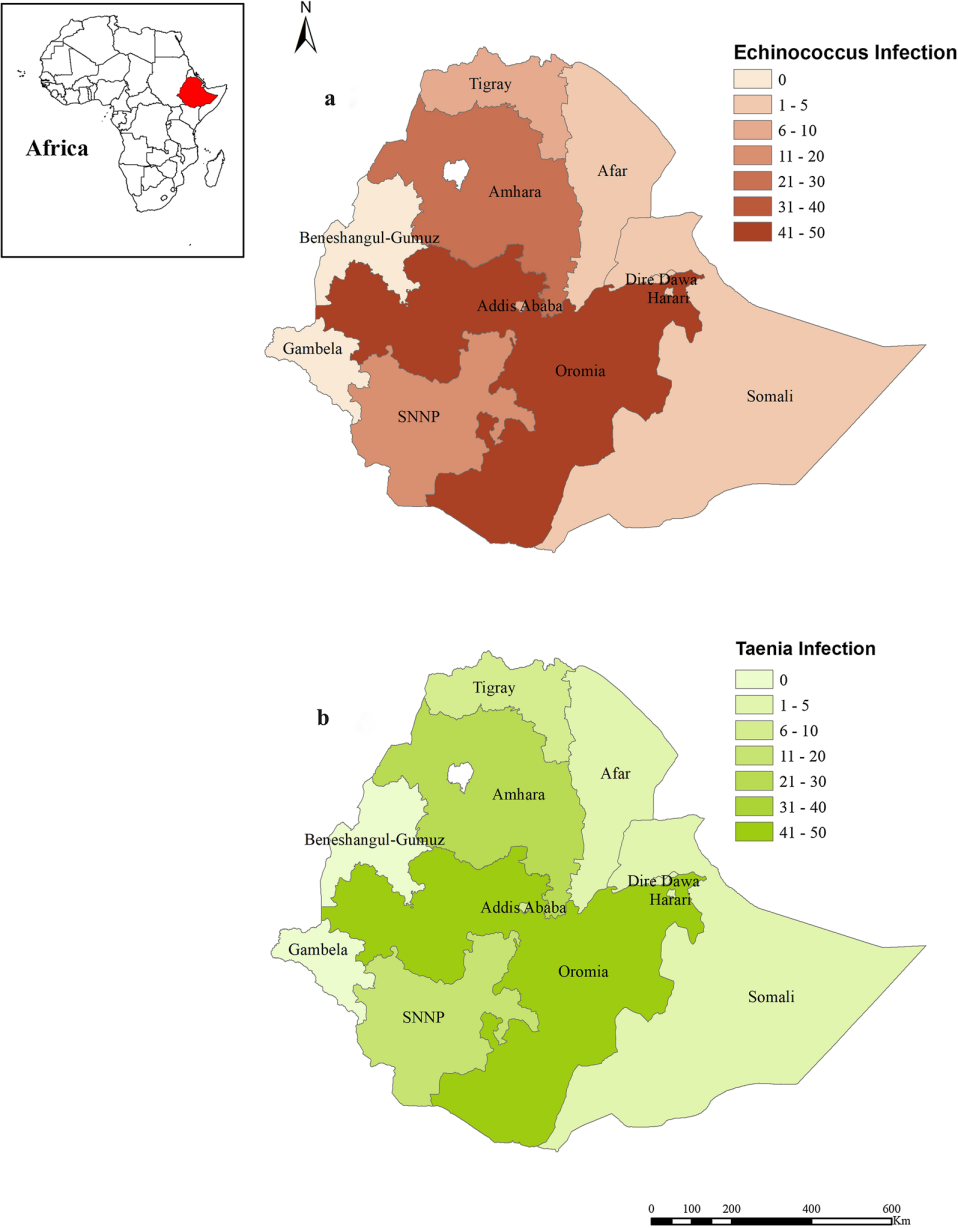
Map of Ethiopia showing regions with study distributions/concentration **a** *Echinococcus* infection, **b** *Taenia* infection. Shapefiles for Ethiopia were retrieved from https://africaopendata.org/dataset/ethiopia-shapefiles and the program ArcMap 10.1 of ArcGIS was used to create the distribution map

*Taenia* and/or *Echinococcus* infections were reported in seven regional states and two chartered cities. However, we could not find reports on *Taenia* and/or *Echinococcus* infections in two regions, namely Gambela and Benishangul-Gumuz regional states. Regarding the regional distribution of reviewed studies, most (32.8%, 66/201) included in this review were from Oromia followed by Amhara (22.9%, 46/201) regional states as indicated in Fig. 2. Furthermore, the number of reports focusing on *Echinococcus* and *Taenia* infections is also shown on the Ethiopian map (Fig. 3); most of the reports are from Oromia regional state. However, the map does not show those reports that were not described by region.

### Human *Taenia*/*Echinococcus* infections

Of the studies included in the systematic review, 58 reported human taeniasis and/or cystic echinococcosis (Table 1). Comparatively, many reports (30.5%) were reviewed from Addis Ababa, the capital of the country. Most data were separately extracted from cross-sectional studies containing active hospital/health facilities reports (*n* = 24) followed by case reports (*n* = 23). Regarding the diagnostic methods used, most (59.3%) of the reports were based on parasitological examination (faecal examination for taeniasis; postmortem examination for CE) followed by imaging (30.5%) for CE. Of the total, cases were reported as taeniasis (*n* = 35) with prevalence range of 0.05–12.2% and CE (*n* = 24) with prevalence range of 0.066–0.7%. Some papers reported taeniasis together with other parasites. The total sample size throughout the study years was 400,450 study subjects in which 1938 were found positive for *Taenia* and/or *Echinococcus* infection (Additional file 2: Table S2).

**Table 1:** Distribution of data sets by human taeniasis and CE, Ethiopia

Characteristics	Frequency	Percent	References
Regions and city administrations			
Tigray	4	6.8	[28, 41–43]
Oromia	8	13.6	[44–51]
Amhara	11	16.9	[52–62]
SNNP	12	20.3	[63–74]
Addis Ababa	16	30.5	[75–90]
Non-specified by region + immigrants	7	12.1	[91–97]
Sex of study participants			
Female	12	20.3	[41, 53, 76, 80, 85, 88, 90, 93–97]
Male	4	6.8	[72, 75, 91, 92]
Both female and male	42	71.2	[28, 42–52, 54–57, 59–71, 73, 74, 77–79, 81–84, 86, 87, 89]
Not reported	1	1.7	[58]
Study report			
Case report	23	39	[28, 41, 53, 72, 75–77, 79–81, 83, 85–88, 90–97]
Retrospective study	11	18.6	[43, 44, 46, 49, 50, 56, 60, 62, 82, 84, 89]
Active hospital/clinic data (on spot)	24	40.7	[42, 47, 48, 51, 52, 54, 55, 57–59, 61, 63–71, 73, 74, 78, 84]
Not indicated	1	1.7	[45]
Diagnostic method used			
Parasitology/faecal examination	34	57.6	[42–48, 50–52, 54, 55, 57–69, 71, 73, 74, 78, 84, 89, 96, 97]
Imaging (ultrasound, CT, x-ray)	18	30.5	[41, 49, 56, 70, 72, 75–77, 81–83, 86–88, 91–93, 95]
Surgery	7	11.9	[28, 53, 79, 80, 85, 90, 94]
Disease category (based on the parasite)			
Taeniasis	35	59.3	[42–48, 50–52, 54, 55, 57–69, 71, 73, 74, 78, 84, 89, 94, 96, 97]
CE	24	40.7	[28, 41, 49, 53, 56, 70, 72, 75–77, 79–83, 85–88, 90–93, 95]

Most human CEs were reported as case reports with a total sample size of 514. These reports focused on unusual presentations and complications of cystic echinococcosis, such as tibial, hepatic, breast, neck, thigh, intra-abdominal, chest wall, cerebral, ovarian, pulmonary, interventricular septum, vertebral, pelvic, and epidural and paraspinal thoracic cyst disease related to *Echinococcus*. There were limited studies focusing on population-based assessments of human CE in Ethiopia. During the study period, only one article showed a prevalence of 0.7% (7/990, 95% CI 0.02–1.20) in a human CE population-based active survey in SNNP, Ethiopia (Additional file 2: Table S2).

### Animal intermediate hosts of *Taenia*/*Echinococcus* infections

A total of 144 data set reports were eligible for the final systematic review focusing on cattle, sheep, goats, camels and pigs. The maximum number of reports by region was conducted in Oromia followed by Amhara regional states. Moreover, a single report may include one or more study regions/city administration. Abattoir-based postmortem examination was frequently used for the detection of these parasite infections; molecular and coprology techniques were also used (Additional file 3: Table S3).

The total sample size throughout the study years was 1,658,057 animals, and 179,722 of them were found positive for *Taenia* and/or *Echinococcus* infections. The most extensive study regarding sample size employed was 1,083,575 animals (goats) while the smallest study included was only 25 animals (camels). The studies reported on bovine, ovine, caprine, camel and swine CE with prevalence range of 2.6–65.15%, 1.1–68%, 0–65%, 12–61.6% and 9.96%, respectively (Additional file 4: Table S4, Additional file 5: Table S5, Additional file 6: Table S6, Additional file 7: Table S7, Additional file 8: Table S8).

Similarly, the studies also reported on bovine cysticercosis with prevalence range of 0.78–30.7%. However, there were only limited reports focusing on the prevalence of *Taenia* and *Echinococcus* infections in some animals. For instance, only one study reported the prevalence of 0.4% (1/257, 95% CI 0.01–2.15) of taeniasis in cattle based on coprological examination; however, the results did not indicate the species level (Additional file 4: Table S4).

The prevalence of *T. hydatigena*, *T. ovis* and *T. multiceps* in sheep across the studies showed prevalence range of 5.73–79%, 2.86–26% and 2.6–19.09%, respectively (Additional file 5: Table S5). Furthermore, the prevalence of *T. hydatigena*, *T. ovis* and *T. multiceps* in goats across the studies showed prevalence range of 8.07–72.38%, 2.1–22% and 0.52–11.7%, respectively (Additional file 6: Table S6).

Direct financial losses associated with organ condemnation reported for some of the parasites were substantial. Some surveys focused on organ condemnation to investigate the financial losses due to infection and other factors that makes calculation of the prevalence difficult. Overall, about 65 studies reported financial losses that range from 4380 ETB or $200 [98] to 34,927,200 ETB or $1,591,216.40 [99] per abattoir survey. The losses were due to the condemnation of edible carcasses and offal such as liver, lung, heart, kidney, spleen, tongue and head. Such reports highlighted the importance of these parasitic infections to the livestock sector.

However, only a few molecular studies (*n* = 7) aimed at identifying and genotyping *Echinococcus* genotypes/species responsible for CE and *Taenia* species were reported (Table 2), which made determining the prevalence of *Echinococcus* species that occur in Ethiopia difficult. At least one molecular study was conducted in all regions except the Amhara, Afar, Benshangul-Gumuz and Gambella regions.

**Table 2:** Molecular studies included in the systematic review and meta-analysis, Ethiopia

Region reported	Host (no. of sampled animals)	Source of sample	Result/molecular identified spp/genotype	References
Oro, DD, Som, AA	Cattle (41)	Cysticerci	*T. saginata* (38/41, 92.7%) Unidentified (7.3%)	[100]
Som, Oro, AA	Sheep (11) Cattle (16) Camel (16)	*Echinococcus* cyst	*E. granulosus* s.s. (87.5) *E. canadensis* (12.5)	[101]
AA, Oro and Tigray	Cattle and sheep	*Echinococcus* cyst	*E. granulosus* s.s, G1 (*n* = 26 samples)	[102]
			*E. granulosus* s.s, G1, *E. ortleppi*, *E. canadensis* G6/7 (*n* = 21 cyst of cattle)	
Har, DD, Oro	Cattle (891) Sheep (95) Goat (95) Camel (25)	*Echinococcus* cyst	*E. granulosus* s.s. (*n* = 165) *E. ortleppi* (*n* = 6) *E. canadensis* G6/7 (*n* = 4)	[103]
Oromia	Cattle Goat Camel Pigs	*Echinococcus* cyst	*E. granulosus* s.s. G1 (*n* = 115, 83.9%) *E. ortleppi G5* (*n* = 6, 4.4%) *E. intermedius* G6/7 (*n* = 16, 11.7%)	[104]
SNNP	Human (1)	*Echinococcus* cyst	*E. granulosus* s.s. as the genotype G_Omo_	[105]
Ethiopia	Spotted hyena (11)	*Taenia* spp.	Demonstrate *T. crocutae* is sister to *T. saginata* and *T. asiatica* whereas *T. solium* was confirmed to be sister to the brown bear tapeworm, *T. arctos*	[72]

### Animal final hosts of *Taenia*/*Echinococcus* infections

Of the studies included in the systematic review, 12 reports discussed the role of prevalence and distribution of *Taenia* and *Echinococcus* infections across the country. As described in Table 3, most (81.8%) reports employed parasitological (faecal and postmortem) examination as diagnostic method and 45.5% investigated adult *Echinococcus* species. The results demonstrated limited employment of molecular techniques for the diagnosis of these parasitic infections in the final hosts. The total sample size throughout the study years was 796 final hosts (dogs, wolf, hyena), and 39.07% were positive either for *Taenia* or *Echinococcus* species.

**Table 3:** Distribution of data sets by animal final hosts’ taeniasis and CE, Ethiopia

Characteristics	Frequency	Percentage	References
Region			
Tigray	2	16.67	[28, 106]
Oromia	3	25	[107–109]
Amhara	3	25	[21, 110, 111]
SNNP	2	16.67	[112, 113]
Oromia + Tigray	1	8.33	[114]
Non-specific	1	8.33	[105]
Study animal			
Dog	9	75	[21, 28, 106, 107, 110–114]
Wolf	2	16.67	[108, 109]
Hyena	1	8.33	[105]
Sex of study animals			
Both	5	41.67	[28, 106, 107, 110, 111]
Unidentified	7	58.33	[21, 105, 108, 109, 112–114]
Diagnostic method^a^			
Parasitological (faeces)	3	23.07	[108, 111, 112]
Parasitological (postmortem)	8	61.54	[21, 28, 106, 107, 110, 111, 113, 114]
Molecular	1	7.69	[105]
Molecular + parasitological	1	7.69	[109]
Parasite type^a^			
Adult *Echinococcus* species	6	35.3	[28, 107, 110, 111, 113, 114]
*T. hydatigena*	2	11.8	[106, 111]
*T. ovis*	2	11.8	[106, 111]
*T. multiceps*	1	5.9	[106]
Unidentified *Taenia* species	6	35.3	[21, 105, 108, 109, 111, 112]

^a^A single paper reports different diagnostic methods and parasite types

In contrast, according to the available data from three regional states, namely Amhara, SNNP and Tigray, average prevalence of taeniasis in dogs was found to be 45.01% with 95% CI as low as 2.1% and as high as 97.3% reporting adult *Taenia* species namely *Taenia hydatigena*, *T. multiceps* and *T. ovis*. In particular, postmortem findings of 51 dogs revealed 56.86% prevalence of taeniasis. The available evidence also indicated a prevalence range of 16.7–88.9% for canine echinococcosis (Additional file 9: Table S9).

### Meta-analysis

The results of the meta-analysis (forest plots, DOi plot and pooled prevalence with their respective 95% CI) and potential publication bias (funnel plots) were separately recorded.

### Overall prevalence of *Taenia* and *Echinococcus* infections

**Fig. 4:**
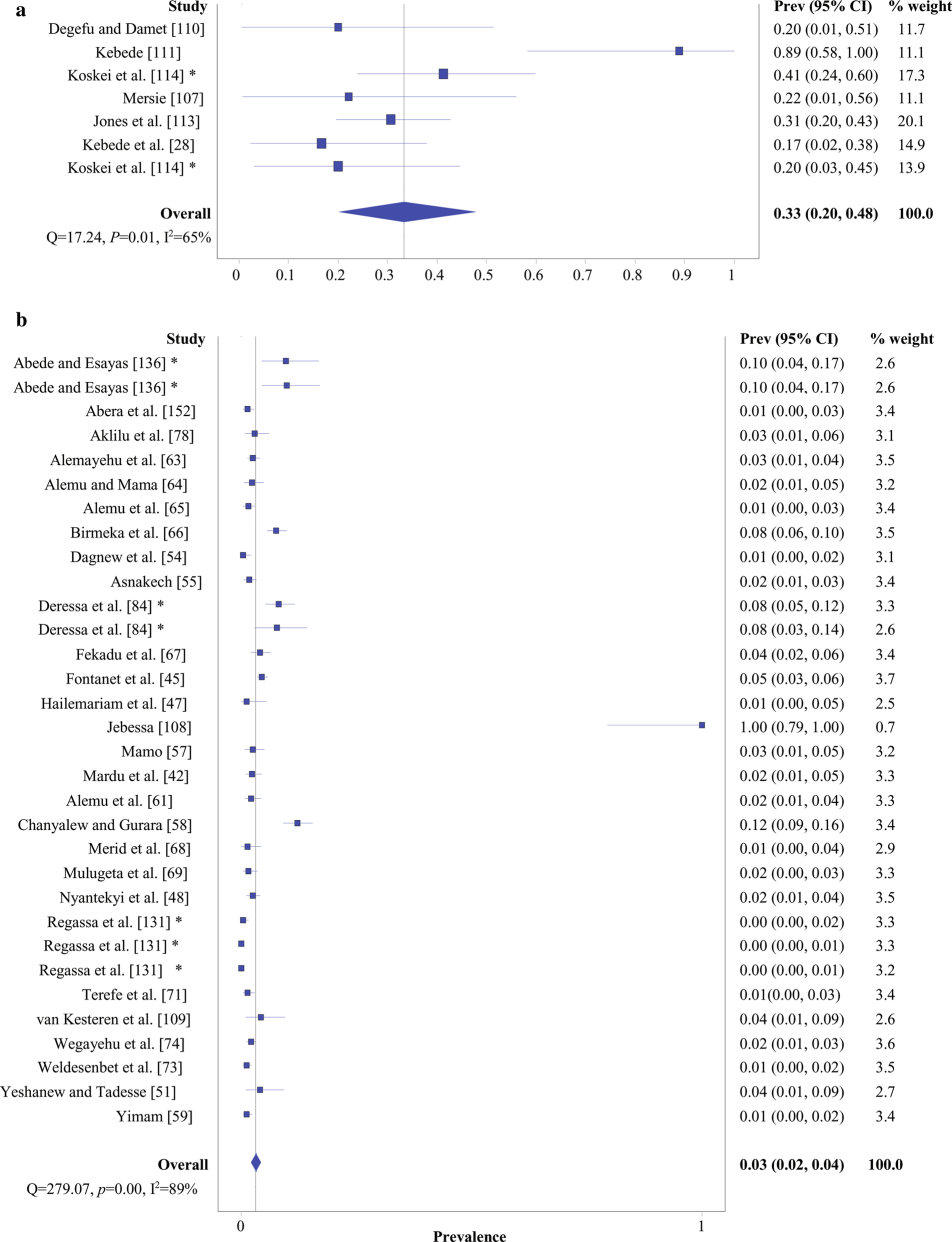
Overall prevalence evidenced by forest plot: **a** dog echinococcosis; **b** taeniasis. *Prev* prevalence, *CI* confidence intervals; *same study

**Fig. 5:**
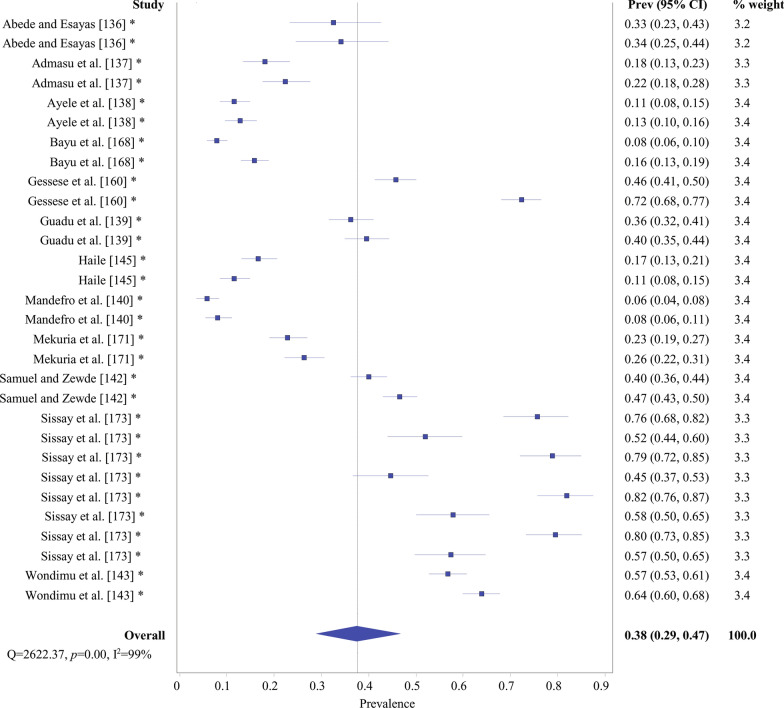
Overall prevalence of *Taenia hydatigena* evidenced by forest plot. *Prev* prevalence, *CI* confidence interval; *same study

**Fig. 6:**
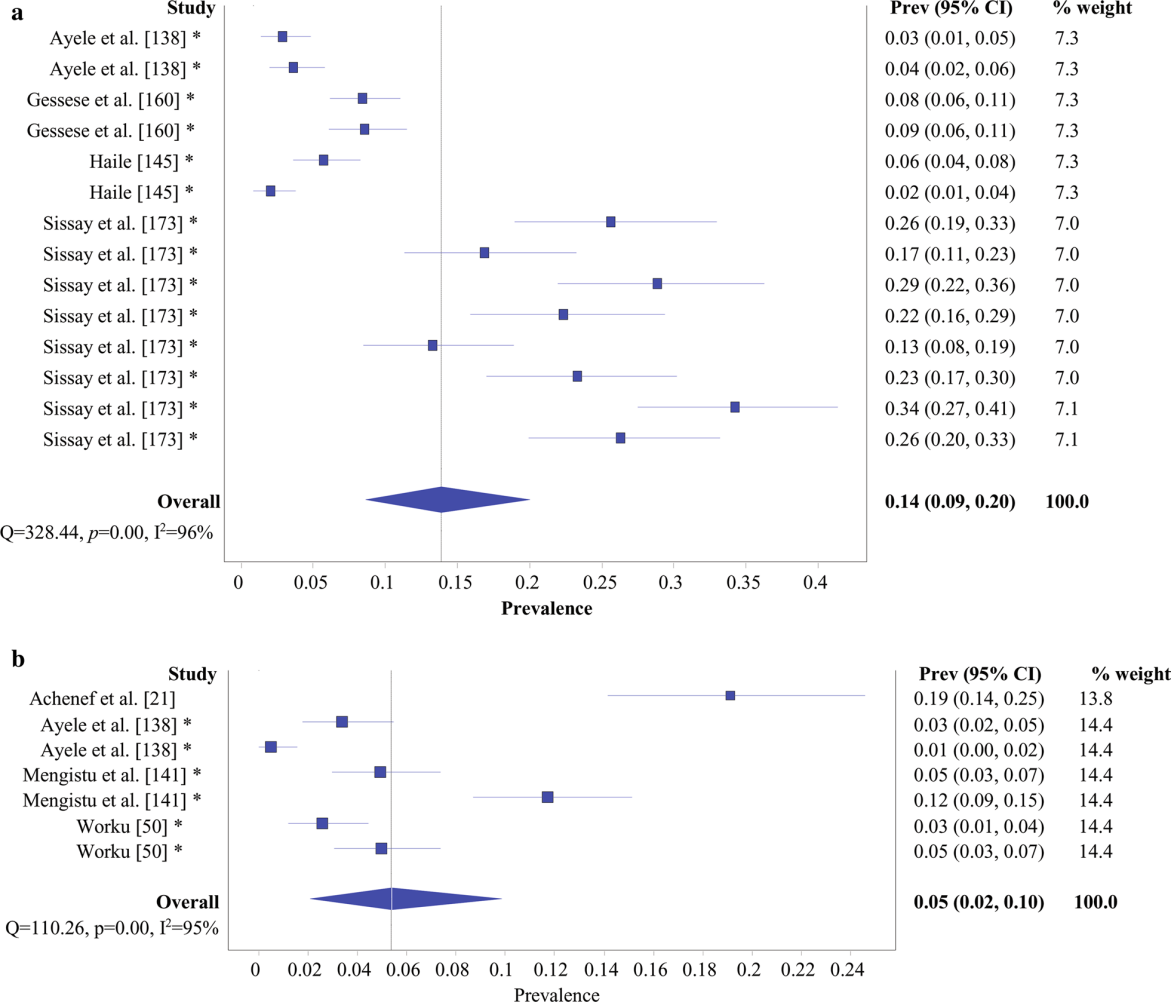
Overall prevalence evidenced by forest plot: **a** *Taenia ovis*; **b** *Taenia multiceps.* *Prev* prevalence, *CI* confidence interval; *same study

From the quantitative analysis, a total of 29,163 intermediate and final hosts were infected by *Taenia* and/or *Echinococcus* [115–173]. Dataset results, pooled effect and heterogeneity are summarized in Table 4. Of the total, 17,971 intermediate hosts were infected by CE with pooled prevalence of 22% (95% CI 18–26%) and high study variability (*Q* = 24,420.65, *I*^2^ = 100%, *P* = 0.000). Moreover, a total of 49 final hosts were infected by echinococcosis with pooled prevalence of 33% (95% CI 20–48%) and between-study variability was low (*Q* = 17.24, *I*^2^ = 65%, *P* = 0.001). Similarly, of the total, 350 study subjects including humans, cattle, sheep, goats and wolves with *Taenia* infection showed a pooled prevalence of 3% (95% CI 2–4%) and between-study variability of (*Q* = 279.07, *I*^2^ = 89, *P* = 0.000). Meanwhile, 3764, 443 and 151 of intermediate hosts were infected by larval stages of *T. hydatigena*, *T. ovis* and *T. multiceps* with pooled prevalence of 38%, 14% and 5%, respectively. The overall prevalence of these parasitic infections is illustrated by forest plots (Figs. 4, 5, 6, Additional file 10: Figure S1).

**Table 4:** Overall pooled prevalence of *Taenia* and *Echinococcus* infections in intermediate and final hosts, Ethiopia

Characteristics	Number of dataset	Pooled effect			Heterogeneity			
		Sample size	Infected	Prevalence (%)	95% CI	Cochran’s *Q*	*I*^2^ (%)	*P*-value
CE	111	96,940	17,971	22	18–26	24,420.65	100	0.000
Cattle	64	52,081	14,248	25.5	22.2–28.9	4829.374	98.695	0.000
Sheep	24	15,585	2209	18.8	13.0–25.4	1908.651	98.795	0.000
Goat	19	26,842	864	13.2	6.3–21.9	2949.349	99.390	0.000
Camel	2	1191	618	47.7	20.4–75.7	83.501	98.802	0.000
Pig	1	251	25	9.96	6.55–14.35	–	–	–
Human	1	990	7	0.7	0.02–1.2	–	–	–
Echinococcosis								
Dog	7	152	49	33	20–48	17.24	65	0.001
Taeniasis	32	10,504	350	3	2–4	279.07	89	0.000
Human	25	9462	319	3.0	2.1–4.0	159.229	84.927	0.000
Cattle	1	257	1	0.4	0.01–2.15	–	–	–
Sheep	2	347	9	3.0	0.00–17.5	23.472	95.740	0.000
Goat	2	336	9	3.1	0.00–17.7	23.225	95.694	0.000
Wolf	2	102	12	51.3	0.00–100	43.568	97.705	0.000
*T. saginata (C. bovis)*								
Cattle	53	111,084	6435	7	5–9	4458	99	0.000
*T. hydatigena*	30	10,561	3764	38	29–47	2622.37	99	0.000
Sheep	15	5238	1816	38.9	25.6–53.0	1464.290	99.044	0.000
Goat	15	5323	1948	36.1	24.6–48.4	1151.815	98.785	0.000
*T. ovis*	14	3753	443	14	9–20	328.44	96	0.000
Sheep	7	1933	244	14.6	6.8–24.6	176.303	96.597	0.000
Goat	7	1820	199	12.6	5.7–21.5	149.389	95.984	0.000
*T. multiceps*	7	2541	151	5	2–10	110.26	95	0.000
Sheep	4	1373	84	5.9	1.5–12.5	53.149	94.356	0.000
Goat	3	1168	67	4.8	0.00–12.4	56.501	96.460	0.000

Furthermore, the pooled prevalence of these cestode infections is presented separately by host in Table 4. Regarding CE, high pooled prevalence of 47.7% (95% CI 20.4–75.7%) in camels followed by 25.5% (95% CI 22.2–28.9%) in cattle, 18.8% (95% CI 13.0–25.4%) in sheep and 13.2% (95% CI 6.3–21.9%) in goats was recorded. For the case of taeniasis, high pooled prevalence of 51.3% (95% CI 0.00–100%) was recorded in wolf followed by goat 3.1% (95% CI 0.00–17.7%).

**Fig. 7:**
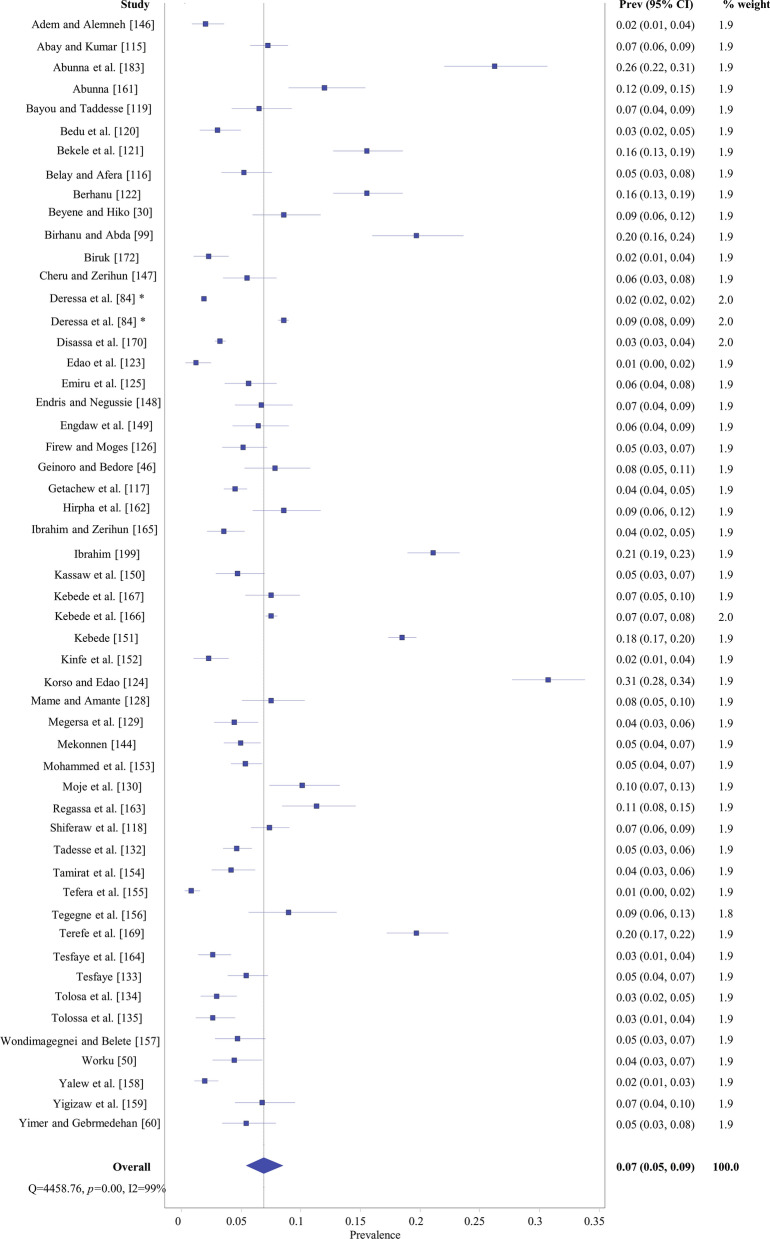
Overall prevalence of *Taenia saginata* (*Cysticercus bovis)* evidenced by forest plot. *Prev* prevalence, *CI* confidence interval; *same study

The random effect meta-analysis of *T. saginata* (bovine cysticercosis) showed that individual study prevalence estimates ranged from 0.78 to 30.7% with an overall pooled prevalence of 7% (95% CI 5–9%). Studies’ weights ranged from 1.8 to 2.0%. Between-study variability was high of (*Q* = 4458.76; *I*^2^ = 99% with a *P*-value of 0.000) (Fig. 7).

However, high prevalences of 38.9% (95% CI 25.6–53.0%), 14.6% (95% CI 6.8–24.6%) and 5.9% (95% CI 1.5–12.5%) of *T. hydatigena*, *T. ovis* and *T. multiceps*, respectively, were reported in sheep (Additional file 5: Table S5).

### Prevalence of *Taenia* and *Echinococcus* infections by region

Significant differences in prevalence of *Taenia* and *Echinococcus* infections between study regions or livestock type have been reported (Additional file 11: Table S10). Region-based group analysis showed 30.1% pooled prevalence of bovine CE at Oromia (95% CI 22.5–38.3%) and 28.4% at Addis Ababa (95% CI 18.5–39.4%). Similarly, high pooled prevalences of 16.6% (95% CI 9.3–25.5%) and 7.4% (95% CI 2.4–14.5%) of ovine and caprine CE, respectively, were recorded in Oromia regional state. Moreover, a pooled prevalence of 37.1% (95% CI 22.4–53.0%) dog echinococcosis was also recorded from the same region. The reports within each of the subgroup of the study animals were highly heterogeneous for CE (*I*^2^ > 96%) except for bovine and ovine CE in Dire Dawa and Amahara, respectively. There was also less heterogeiniety in reports of dog echinococcosis in Amhara, Oromia and Tigray regional states (*I*^2^ < 90%).

Based on coprological results, comparatively high pooled prevalence of human taeniasis was recorded in Addis Ababa (4.0%, 95% CI 1.1–8.3%) followed by Oromia (3.9%, 95% CI 2.4–5.6%). Meanwhile, two research papers with pooled prevalence of 51.3% (95% CI 0.00–100%) of taeniasis in wolves (*Canis simensis*) were documented from Oromia regional state.

Most cattle were found to be infected by *T. saginata* in SNNP with a pooled prevalence of 10.5% (95% CI 4.0–19.4%) followed by 7.9% (95% CI 5.7–10.4%) in Oromia regional state. Regarding *T. hydatigena*, high prevalence was reported from Dire Dawa with a pooled prevalence of 52.8% (95% CI 0.00–100%) and 40.7% (95% CI 10.5–74.6%) in sheep and goat, respectively. Likewise, a pooled prevalence ranging from 3.6 to 8.3% of *T. ovis* and *T. multiceps* from both sheep and goat was reported from Oromia regional state. Additional file 11: Table S10 presents the summary of key findings reported in this review.

### Publication bias

The existence of publication bias was assessed in some study groups but not for some because of the nature of the article (case report) or not enough publication data to discuss the possible impact on infection prevalence (camel, pig, dog, wolf and hyena). Possible publication bias was demonstrated by visualization of asymmetry in funnel plots with their respective LFK values. Accordingly, there was no asymmetry for echinococcosis (LFK index = 0.23), taeniasis (LFK index = 0.93), *T. hydatigena* (LFK index = 0.61) and *T. multiceps* (LFK index = 0.58) infection. There was minor asymmetry for hydatidosis (LFK index = 1.59). In contrast, there was major asymmetry for *T. ovis* (LFK index = 2.59) and *T. saginata* (LFK index = 3.07), which indicates the presence of publication bias as indicated in the funnel plots presented (Additional file 12: Figure S2, Additional file 13: Figure S3, Additional file 14: Figure S4, Additional file 15: Figure S5, Additional file 16: Figure S6, Additional file 17: Figure S7, Additional file 18: Figure S8).

## Discussion

This SR and meta-analysis summarized the current evidence on the prevalence and distribution of *Taenia* and *Echinococcus* infections in Ethiopia. More than 15,500 potentially relevant references were assessed, a fact that emphasizes the broad relevance of the topic.

At least one report was documented from each region and chartered cities except for Gambela and Benishangul-Gumuz. The absence of data, however, does not exclude the existence of cestode infection in these regions. Moreover, poor diagnosis and reporting, particularly in rural areas, indicate that the data accrued are likely to underestimate occurrence.

Abattoir-, hospital-, household- and field-based studies conducted across the country were used as source of data. Most of the studies were conducted in Oromia and Amhara followed by Addis Ababa and SNNP regional states. The geographical distribution of *Taenia* and *Echinococcus* infection within the country was uneven and might affect the generalization of the findings. Spatial variation in the infection prevalence in livestock was previously reported by Jobre et al. [174] and Kebede et al. [175]. The distribution may also be influenced by temperature and humidity [176].

Most human CE was reported as case reports focused on unusual presentations and complications of cystic echinococcosis, such as tibial, hepatic, breast, neck, thigh, intra-abdominal, chest wall, cerebral, ovarian, pulmonary, interventricular septum, vertebral, pelvic, and epidural and paraspinal thoracic cysts, which makes them non-representative of the epidemiology of cystic echinococcosis in the affected area [177]. Evidence from hospital-based case reports from other parts of the world indicates that the condition of patients with cerebral *Echinococcus* cysts depends on the size and location of the cysts. However, the lack of advanced imaging techniques in most rural health facilities in sub-Saharan Africa where the disease is endemic could, in part, contribute to the lack of reported cases of cystic echinococcosis [178]. Similarly, population-based active surveys have not been well documented and explored at the country level. This may be attributed to the fact that it is a neglected disease [179]. In addition, the diagnosis requires advanced techniques for confirmation particularly in humans [180]. Furthermore, five studies were included in this study where the diagnosis was made in Ethiopian immigrants outside the country. This indicates the chronic nature of the disease [181, 182].

The high prevalence of taeniasis in most developing countries including Ethiopia is due to the habit of consuming raw or undercooked beef [183, 184]. In the current study, high prevalence of taeniasis based on microscopy was found in Addis Ababa followed by Oromia region. However, coprological techniques have fairly low sensitivities associated with intermittent egg excretion and depend on the technique used [185, 186].

Most studies in animal intermediate hosts were conducted in Oromia and Amhara regions because of their infrastructure advantages (both municipal and private abattoirs). Similarly, most of the hosts investigated were ruminants (cattle, sheep and goat) because of the high meat demand of the people inhabiting these regions. This will have a direct impact on the life cycle of *Taenia* and *Echinococcus* spp. [187]. The life cycle is completed when the final host, such as dogs, ingests *Echinococcus* cysts containing protoscolices [188] where there is enough access to visceral organs from the slaughtered animals.

In the current study available evidence suggests limited application of molecular tools. This is because little funding is given for research and management of neglected tropical diseases in sub-Saharan Africa [189, 190]. Another reason could be the lack of well-equipped laboratories and trained personnel.

Though the life cycle of taeniasis and echinococcosis involves canids, limited studies have been conducted at different interfaces. For instance, there are > 55 protected areas (including 21 national parks) in the country [191] where potential intermediate and final hosts are present, but the status of the infections is not well reported. However, the interaction between wildlife and livestock transmitted forms is likely to have an impact on human and animal health in the vicinity of the national parks [102].

Regarding livestock, several cystic echinococcosis investigations in cattle from several parts of Ethiopia have found regional differences in prevalence. Similar studies in Kenya, a neighboring country, demonstrated that cystic echinococcosis occurs in most parts of the country but available data are mostly from Turkana communities in the northwest and from Maasai communities in the south [177]. The current study also showed similar variation in the prevalence of CE among the different regions of the country. Similarly, Omer and her colleagues [192] also documented similar findings in Sudan. From central Sudan, prevalence ranged from 20% (cattle) to 55.6% (camels). In western Sudan, prevalence is highest among camels (61.4% of 565) followed by sheep (11.9% of 9272). In southern Sudan, varying prevalences in cattle (7.1% of 325), sheep (2.7% of 295) and goats (7.1% of 42) have also been reported.

In Africa, though differing by country and also region, CE is reported most commonly in cattle [193–195]. Meanwhile, CE is the major cause of organ condemnation in most Ethiopian abattoirs and leads to huge economic losses [196, 197]. In the present review higher pooled prevalence of CE was recorded in camels and cattle than sheep and goats. Variation among the intermediate hosts could be ascribed to the age factor. For instance, cattle and camels are generally slaughtered at older age than sheep and goats and consequently are exposed to infection over a longer period of time [198]. Ibrahim [199], Cabrera et al. [200] and Guorino et al. [201] also reported an increase in prevalence of the disease with increase in age.

The low prevalence of *Echinococcus* cyst infection in pigs in Ethiopia could be mainly attributed to the absence of extensive swine farms in the country [198]. Furthermore, pork is not consumed by most of people in Ethiopia. In general, information on echinococcosis in pigs in sub-Saharan Africa is scarce [177] but high prevalence (56%) is reported from West Africa in the region the Niger Delta [202]. In the current review, though reports are limited, the findings showed the significance of dog echinococcosis. Globally, echinococcosis presents a serious health concern especially in endemic countries [31, 32] where transmission of the disease is affected by the prevalence of the parasite in domestic dogs, behaviors of humans towards dogs and other related factors [203].

There are considerable variations in the prevalence and distribution of *T. saginata* (bovine cysticercosis) in different areas that are not easily explained by the existing information. The overall pooled prevalence was lower than in reports from Nigeria, 29% [204], but higher than in reports from Kenya, 2.56% [205], whereas the prevalence ranged from 0.2 to 20% in Egypt [206]. Results showed a widespread occurrence of metacestodes in sheep and goats in Ethiopia where *T. hydatigena* (*C. tenuicollis*) was the most common cestode (metacestode) reported, in line with the report of Asmare et al. [207].

Studies to determine the prevalence of coenurosis (*T. multiceps*) in small ruminants show low variation in their results. In contrast, this result is much lower than the findings of Desouky et al. [208] and Miran et al. [209] in Tanzania who reported a *T. multiceps* prevalence of 44.4% in small ruminants (45.6% in sheep and 43.3% in goats). This could be due to factors that play an important role in the epidemiology of *T. multiceps* [210].

Most taeniids of dogs are globally distributed. In the current study, only two studies described the status of *Taenia* infection with average prevalence of 45% of *T. hydatigena*, *T. multiceps* and *T. ovis*. This finding is supported by Mulinge et al*.* [211] who found *T hydatigena* and *T. multiceps* were the most frequent taeniids of dogs in some parts of Kenya. Such findings demonstrate the involvement of dogs in the transmission cycles of the diseases. However, the limited number of studies (only two reports) from most of the regions affected the reflection of the real situation of these parasites in the country.

Molecular studies during the survey period covered most of the regions, though the numbers of studies are unbalanced, which made determining the prevalence of *Echinococcus* species that occur in Ethiopia difficult; they ranged from one to five reports per region, except for Afar, Amhara, Benishangul-Gumuz and Gambela regional states where molecular data were absent. These reports showed the presence of different genotypes, *E. ortleppi* (genotype 5), *E. granulosus* s.s., *E. canadensis* and *E. intermedius* (genotype 6). This shows cattle may have an important role in the life cycle of this disease and indicates the existence of potential transmission to human and other susceptible hosts [212–214]. Moreover, genotype 6 has been identified in human CE in Argentina, Nepal and Iran [215–217] cited by [181].

### Study limitation

We observed study limitations such as the scantiness of data in some regions, publication bias, heterogeneity between studies and the uneven prevalence distribution among study regions. Few reports had results showing tapeworm infection (broad classification) that were not identified at the species level and some studies reported taeniasis together with other parasites. Moreover, only few studies reported at genotype level, which made determining the prevalence of *Echinococcus* spp. that occur in Ethiopia difficult. Lastly, when the meta-analysis included only a small number of studies, it was not possible to assess publication bias using funnel plots.

## Conclusion

In the current study, we provided comprehensive information on the prevalence and distribution of *Taenia* and *Echinococcus* infections in Ethiopia. The results showed the status of these cestode infections in different regions, but studies were mainly carried out around central Ethiopia, particularly Oromia and Addis Ababa, because of the presence of relatively more infrastructure. The meta-analysis confirmed a high degree of variability in pooled prevalence of these parasitic infections. However, there are still many data gaps with respect to the research coverage and agro-ecological factors contributing to the parasite prevalence and distribution across the country, which urges further studies. Therefore, annual surveillance of infection rates in dogs, livestock and humans is critical for determining a pre-intervention baseline and evaluating the effectiveness of control programmes.

## Supplementary Information

**Additional file 1: Table S1.** PRISMA 2009 checklist.

**Additional file 2: Table S2.** Characteristics of studies included in the systematic review and meta-analysis (study subject: human). F, female; M, male; B = both male and female; Imm, immigrant; CS, cross sectional; p, prevalence; CI, confidence interval.

**Additional file 3: Table S3.** Distribution of data sets by animal intermediate hosts’ taeniasis and CE, Ethiopia. n, number of report; AA, Addis Ababa; Oro, Oromia; Tig, Tigray; SNNP, Southern Nation and Nationality of People; Amh, Amhara; Har, Harar; DD, Dire Dawa; Som, Somali; *some papers reported more than one parasite hence multiple datasets.

**Additional file 4: Table S4.** Characteristics of studies included in the systematic review and meta-analysis (study subject: cattle). F, female; M, male; B = both male and female; CS, cross sectional; p, prevalence; CI, confidence interval.

**Additional file 5: Table S5.** Characteristics of studies included in the systematic review and meta-analysis (study subject: sheep). F, female; M, male; B = both male and female; CS, cross sectional; p, prevalence; CI, confidence interval.

**Additional file 6: Table S6.** Characteristics of studies included in the systematic review and meta-analysis (study subject: goat). F, female; M, male; B = both male and female; CS, cross sectional; p, prevalence; CI, confidence interval.

**Additional file 7: Table S7.** Characteristics of studies included in the systematic review and meta-analysis (study subject: camel). F, female; M, male; B = both male and female; CS, cross sectional; p, prevalence; CI, confidence interval.

**Additional file 8: Table S8.** Characteristics of studies included in the systematic review and meta-analysis (study subject: pig). F, female; M, male; B = both male and female; CS, cross sectional; p, prevalence; CI, confidence interval.

**Additional file 9: Table S9.** Characteristics of studies included in the systematic review and meta-analysis (study subject: final hosts). F, female; M, male; B = both male and female; CS, cross sectional; p, prevalence; CI, confidence interval.

**Additional file 10: Figure S1.** Overall prevalence of cystic echinococcosis evidenced by forest plot. Prev, prevalence; CI, confidence interval; *same study; **same name of first authors.

**Additional file 11: Table S10.** Pooled prevalence of *Taenia* and *Echinococcus* infections in intermediate and final hosts by region, Ethiopia.

**Additional file 12: Figure S2.** Publication bias evidenced by funnel plots for overall prevalence of cystic echinococcosis. Prev, prevalence.

**Additional file 13: Figure S3.** Publication bias evidenced by funnel plots for overall prevalence of dog echinococcosis. Prev, prevalence.

**Additional file 14: Figure S4.** Publication bias evidenced by funnel plots for overall prevalence of taeniasis. Prev, prevalence.

**Additional file 15: Figure S5.** Publication bias evidenced by funnel plots for overall prevalence of *T.saginata* (*C. bovis*). Prev, prevalence.

**Additional file 16: Figure S6.** Publication bias evidenced by funnel plots for overall prevalence of *T. hydatigena*. Prev, prevalence.

**Additional file 17: Figure S7.** Publication bias evidenced by funnel plots for overall prevalence of *T. ovis*. Prev, prevalence.

**Additional file 18: Figure S8.** Publication bias evidenced by funnels plots for overall prevalence of *T. multiceps*. Prev, prevalence.

## Data Availability

All data generated or analysed in this paper are provided as Additional files.

## Abbreviations

AE: Alveolar echinococcosis
CE: Cystic echinococcosis
CI: Confidence interval
ETB: Ethiopian Birr
LFK: Luis Furuya-Kanamori
PRISMA: Preferred Reporting Items for Systematic Reviews and Meta-Analysis
SNNP: South Nation and Nationality of People
SSA: Sub-Saharan African
