# Supplementary material for: A systematic review and meta-analysis on prevalence and distribution of Taenia and Echinococcus infections in Ethiopia
Source: Parasit Vectors. 2021 Sep 6;14:447. doi: 10.1186/s13071-021-04925-w (PMC8419976; doi:10.1186/s13071-021-04925-w)
Supplement: Supplementary file 2 — Additional file 2: Table S2. Characteristics of studies included in the systematic review and meta-analysis (study subject: human). F, female; M, male; B = both male and female; Imm, immigrant; CS, cross sectional; p, prevalence; CI, confidence interval. [file 13071_2021_4925_MOESM2_ESM.doc]

| **Reference** | **Study area** | **region** | **year of study** | | **sex** | **Age (yrs)** | **Study design/data** | **dx method** | **Sample size** | **no +** | **P (%)** | **95% CI** | **Disease category** |
| --- | --- | --- | --- | --- | --- | --- | --- | --- | --- | --- | --- | --- | --- |
| Abdullah et al., 2016 | Ethiopian immigrant in Kuwait | imm | - | - | F | 22 | case report | ultrasound | 1 | 1 | - |  | CE |
| Abebe and Tsehay, 2016 | St. Paul's hospital Millennium Medical college | Addis Ababa | - | - | M | 16 | case report | ultrasound | 1 | 1 | - |  | CE |
| Abebe, 2010 | Menilik II Hospital | Addis Ababa | - | - | F | 25 | case report | x-ray | 1 | 1 | - |  | CE |
| Abebe et al., 2017 | SPHMMC | Addis Ababa | 06, 2016 | 10, 2016 | B | mixed | retrospective, case report | ultrasound | 44 | 44 | - |  | CE |
| Abera et al., 2010 | Bahir Dar Town | Amhara | 04, 2009 | 04, 2009 | B | mixed | CS | parasitological | 384 | 5 | 3.53 | 0.42 -  3.01 | Taeniasis |
| Aderaye, 1998 | Ethiopia | Eth | - | - | M | 28 | case report | x-ray | 1 | 1 | - |  | CE |
| Ajmera and Simon, 2010 | Ethiopian immigrating to the US | imm | - | - | F | 23 | case report | surgery | 1 | 1 | - |  | Taeniasis |
| Aklilu et al., 2015 | Addis Ababa | Addis Ababa | 01, 2013 | 05, 2013 | B | mixed | CS | parasitological | 172 | 5 | 5.2 | 0.95- 6.65 | Taeniasis |
| Alemayehu et al., 2017 | Wolaita zone | SNNP | 01, 2015 | 02, 2015 | B | mixed | CS | parasitological | 503 | 13 | 2.6 | 1.38 -  4.38 | Taeniasis |
| Alemu and Mama, 2017 | Aribaminch | SNNP | 01, 2016 | 08, 2016 | B | mixed | CS | parasitological | 213 | 5 | 2.3 | 0.80 -  5.40 | Taeniasis |
| Alemu et al., 2014 | Umolante district, South Ethiopia | SNNP | 11, 2010 | 01, 2011 | B | mixed | CS | parasitological | 405 | 6 | 1.5 | 0.55 -  3.20 | Taeniasis |
| Ali et al., 2005 | Tikur Anbessa Hospital | Addis Ababa | - | - | B | mixed | case report | surgery | 72 | 72 | - |  | CE |
| Almazeedi et al., 2014 | Ethiopian immigrating to Kuwait | imm | - | - | F | 22 | case report | ultrasound | 1 | 1 | - |  | CE |
| Argaw et al., 2017 | St. Paul’s Hospital Millennium Medical College | Addis Ababa | - | - | F | 36 | case report | surgery | 1 | 1 | - |  | CE |
| Assefa et al., 2011 | Tikur Anbessa and Zewditu teaching hospitals | Addis Ababa | 2009 | 2011 | B | mixed | case series | imaging | 4 | 4 | - |  | CE |
| Assefa et al., 2015 | AA abatoir | Addis Ababa | 10, 2011 | 03, 2012 | B | mixed | retrospective | ultrasound | 25840 | 27 | 0.1 | 0.07 -  0.15 | CE |
| Assefa et al., 2014 | Ethiopia | Eth | - | - | M | 16 | case report | radiology | 1 | 1 | - |  | CE |
| Bekele and Firew, 2016 | Amhara | Amhara | - | - | F | 18 | case report | surgery | 1 | 1 | - |  | CE |
| Biluts et al., 2006 | Tikur Anbessa hospital | Addis Ababa | - | - | B | adults | retrospective, case report | ultrasound | 137 | 137 | - |  | hepatic CE |
| Birmeka et al., 2017 | Gurage Zone | SNNP | - | - | B | mixed | CS | parasitological | 641 | 49 | 7.6 | 5.70 -  9.90 | Taeniasis |
| Chala, 2013 | Mojo Health Center | Oromia | - | - | B | mixed | retrospective | parasitological | 53942 | 29 | 0.05 | 0.04 -  0.08 | Taeniasis |
| Dagnew et al., 2012 | Gondar University | Amhara | 01, 2011 | 06, 2011 | B | mixed | CS | parasitological | 200 | 1 | 0.5 | 0.01 -  2.75 | Taeniasis |
| Asnakech, 2016 | Gorebella health center, Ankober | Amhara | 01, 2016 | 04, 2016 | B | mixed | CS | parasitological | 403 | 7 | 1.7 | 0.70 -  3.60 | Taeniasis |
| Deressa et al., 2012 * | St. Paul Hosp & EHNRI | Addis Ababa | 11, 2009 | 03, 2010 | B | mixed | CS | parasitological | 384 | 31 | 8.1 | 5.55 -  11.26 | Taeniasis |
| Deressa et al., 2012 * | Mojo | Oromia | 11, 2009 | 03, 2010 | B | mixed | CS, retrospective | parasitological | 366 | 24 | 6.6 | 4.25 -  9.60 | Taeniasis |
| Dessie, 2017 | Aykel Health Center, North Gondar | Amhara | - | - | B | mixed | retrospective | parasitological | 36735 | 26 | 0.07 | 0.05 -  0.10 | Taeniasis |
| Fekadu et al., 2013 | Hawassa University Referral Hospital | SNNP | 08, 2008 | 12, 2008 | B | mixed | CS | parasitological | 343 | 14 | 4.1 | 2.25 -  6.75 | Taeniasis |
| Fontanet et al., 2000 | Wonji and Shoa | Oromia | - | - | B | mixed | - | parasitological | 1239 | 56 | 4.5 | 3.40 -  5.80 | taeniasis |
| Gaym et al., 2002 | Tikur Anbessa | Addis Ababa | - | - | F | 35 | case report | surgery | 1 | 1 | - |  | CE |
| Geinoro and Bedore, 2019 | Bishoftu MA | Oromia | 10, 2013 | 04, 2014 | B | mixed | CS, retrospective | parasitological | 84035 | 121 | 0.14 | 0.12 -  0.17 | Taeniasis |
| Hailemariam et al., 2004 | Jimma Hospital | Oromia | 01, 2002 | 02, 2002 | B | mixed | CS | parasitological | 78 | 1 | 1.3 | 0.03 -  6.90 | Taeniasis |
| Kassa et al., 2014 | Mekelle | Tigray | - | - | F | 53 | case report | x-ray | 1 | 1 | - |  | *Echinococcus* cyst |
| Kassa et al., 2009 | Mekelle, Axum, Maichew | Tigray | - | - | B | mixed | case report | parasitological | 9 | 9 | - |  | CE |
| Kebede et al., 2010 | Bahir Dar | Amhara | - | - | B | mixed | retrospective | ultrasonography | 36402 | 24 | 0.066 | 0.04 -  0.10 | CE |
| Nyantekyi et al., 2014 | Abaye Deneba | Oromia | 06, 2008 | 06, 2008 | B | mixed | CS | parasitological | 491 | 12 | 2.4 | 1.30 -  4.20 | taeniasis |
| Mamo, 2014 | Shewa-Robit | Amhara | 11, 2008 | 11, 2008 | B | mixed | CS | parasitological | 236 | 6 | 2.5 | 0.94-5.45 | Taeniasis |
| Mardu et al., 2019 | Mekelle | Tigray | 02, 2017 | 06, 2017 | B | mixed | CS | parasitological | 291 | 7 | 2.4 | 0.97 -  4.89 | Taeniasis |
| Alemu et al., 2019 | Lay Gayint, S Gonder | Amhara | 05, 2016 | 05, 2016 | B | mixed | CS | parasitological | 273 | 6 | 2.2 | 0.80 -  4.70 | Taeniasis |
| Chanyaleu and Gurara, 2014 | Tach Gayint District, South Gondar | Amhara | 11, 2008 | 05, 2009 | ? | mixed | CS | parasitological | 403 | 49 | 12.2 | 9.10 -  15.80 | Taeniasis |
| Merid et al., 2001 | Hawassa | SNNP | 11, 1998 | 11, 1998 | B | mixed | CS | parasitological | 139 | 2 | 1.4 | 0.20 -  5.10 | Taeniasis |
| Mhatebu, 2017 | Asella and Adama | Oromia | 11, 2011 | 03, 2012 | B | mixed | retrospective | ultrasound | 35697 | 67 | 0.2 | 0.15 -  0.24 | CE |
| Minas et al., 2007 | Tikur Anbessa Hospital | Addis Ababa | - | - | B | mixed | CS, retrospective, case report | ultrasound | 234 | 234 | - |  | CE |
| Mulugeta et al., 2019 | Hosanna Town | SNNP | - | - | B | mixed | CS | parasitological | 261 | 4 | 1.5 | 0.40 -  3.90 | Taeniasis |
| Noss et al., 2013 | Ethiopian migrant to US | imm | - | - | F | 23 | case report | parasitological | 1 | 1 | - |  | Taeniasis |
| Klungsoyr et al., 1993 | Hammer | SNNP | - | 1989 | B | mixed | CS | ultrasound | 990 | 7 | 0.7 | 0.02 -  1.20 | CE |
| Shippey et al., 2002 | Ethiopian migrant to US | imm | - | - | F | 22 | case report | parasitological | 1 | 1 | ? |  | Taeniasis |
| Sisay et al., 2015 | Tikur Anbessa Hospital | Addis Ababa | - | - | B | pediatrics | retrospective, case report | ultrasound | 10 | 10 | - |  | CE |
| Tefera et al., 2017 | school of medicine AAU | Addis Ababa | - | - | F | 9 | case report | x-ray | 1 | 1 | - |  | CE |
| Terefe et al., 2011 | Bushulo village | SNNP | 05, 2007 | 06, 2007 | B | mixed | CS | parasitological | 419 | 6 | 1.4 | 0.53 -  3.09 | Taeniasis |
| Tesema, 2011 | Tikur Anbessa hospital | Addis Ababa | 04, 2011 | 06, 2011 | B | mixed | retrospective | parasitological | 4977 | 20 | 0.4 | 0.25 -  0.62 | Taeniasis |
| Wassermann et al., 2016 | Hammer, SNNP | SNNP | - | - | M | 55 | case report | ultrasound | 1 | 1 | - |  | CE |
| Wegayehu et al., 2013 | Gamo area, South Ethiopia | SNNP | 09, 2010 | 07, 2011 | B | mixed | CS | parasitological | 858 | 18 | 2.1 | 1.25 -  3.30 | Taeniasis |
| Weldesenbet et al., 2019 | Gurage zone | SNNP | - | - | B | mixed | CS | parasitological | 600 | 7 | 1.2 | 0.47 -  2.39 | Taeniasis |
| Wester et al., 2016 | Collage of Health Science, AAU | Addis Ababa | - | - | F | 20 | case report | surgery | 1 | 1 | - |  | CE |
| Worku, 2017* | Bishoftu Elfora Export Abattoir | Oromia | 11, 2015 | 03, 2016 | B | mixed | retrospective | parasitological | 74684 | 459 | 0.61 | 0.56 -  0.67 | Taeniasis |
| Yemane and Kumar, 2018 | Mekelle | Tigray | 11, 2013 | 03, 2014 | B | mixed | CS, retrospective | parasitological | 34310 | 193 | 0.56 | 0.49 -  0.65 | taeniasis |
| Yeshanew and Tadesse, 2017 | Mettu | Oromia | 03, 2013 | 06, 2013 | B | mixed | CS | parasitological | 96 | 4 | 4.2 | 1.20 -  10.30 | Taeniasis |
| Yimam, 2016 | Hara Health Center, South Wollo | Amhara | 11, 2015 | 04, 2016 | B | mixed | CS | parasitological | 430 | 5 | 1.2 | 0.40 -  2.70 | Taeniasis |
| Yimer and Gebrmedehan, 2019 | Debre Brihan | Amhara | 12, 2016 | 04, 2017 | B | mixed | CS, retrospective | parasitological | 2484 | 97 | 3.9 | 3.20 -  4.70 | Taeniasis |

*Age: F = female, M= male, B= both male & female; Imm = Immigrant p = prevalence; CS= cross sectional
