# Supplementary material for: A systematic review and meta-analysis on prevalence and distribution of Taenia and Echinococcus infections in Ethiopia
Source: Parasit Vectors. 2021 Sep 6;14:447. doi: 10.1186/s13071-021-04925-w (PMC8419976; doi:10.1186/s13071-021-04925-w)
Supplement: Supplementary file 3 — Additional file 3: Table S3. Distribution of data sets by animal intermediate hosts’ taeniasis and CE, Ethiopia. n, number of report; AA, Addis Ababa; Oro, Oromia; Tig, Tigray; SNNP, Southern Nation and Nationality of People; Amh, Amhara; Har, Harar; DD, Dire Dawa; Som, Somali; *some papers reported more than one parasite hence multiple datasets. [file 13071_2021_4925_MOESM3_ESM.doc]

| **Characteristics** | | **Study subjects** | | | | |
| --- | --- | --- | --- | --- | --- | --- |
|  |  | **Cattle**  **(n =135)** | **Sheep**  **(n = 48)** | **Goat**  **(n = 41)** | **Camel (n = 5)** | **Pig**  **(n = 2)** |
|  |  | **N (%)** | **N (%)** | **N (%)** | **N (%)** | **N (%)** |
| Regions and city administrations | Tigray | 12 (8.9) | 1 (2.1) | - | - | - |
|  | Oromia | 51 (37.8) | 29 (60.4) | 29 (70.7) | 2 (40) | 1 (50) |
|  | Amhara | 37 (27.4 | 8 (16.7) | 3 (7.3) | - | - |
|  | SNNP | 15 (11.1) | - | - | - | - |
|  | Addis Ababa | 6 (4.4) | 4 (8.3) | 4 (9.8) | - | 1 (50) |
|  | Harari | 2 (1.5) | - | - | - | - |
|  | Dire Dawa | 3 (2.2) | 1 (2.1) | 1 (2.4) | - | - |
|  | Afar | - | - | - | 1 (20) | - |
|  | Somali | 1 (0.7) | - | - | - | - |
|  | AA_Oro | 2 (1.5) | - | - | - | - |
|  | Tig_Oro_Amh | 1 (0.7) | - | - | - | - |
|  | Har_DD_Oro | 1 (0.7) | 1 (2.1) | 1 (2.4) | 1 (20) | - |
|  | Som_Oro_AA | 1 (0.7) | 1 (2.1) |  | 1 (20) | - |
|  | Oro_DD_Som_AA | 1 (0.7) | - | - | - | - |
|  | Oro_Har_DD_Som | - | 3 (6.3) | 3 (7.3) | - | - |
|  | Ethiopia (unspecified by region) | 2 (1.5) | - | - | - | - |
| Sex of study animals | Female | 0 (0) | 0 (0) | 0 (0) | 0 (0) | 0 (0) |
|  | Male | 9 (6.7) | 2 (4.2) | - | - | - |
|  | Both female & male | 76 (56.3) | 24 (50) | 21 (51.2) | 2 (40) | 2 (100) |
|  | Not indicated | 50 (37) | 22 (45.8) | 20 (48.8) | 3 (60) | - |
| Age of study animals | Mixed (both young & adult) | 104 (77) | 39 (81.3) | 33 (80.5) | 1 (20) | 1 (50) |
|  | Unspecified | 31 (23) | 9 (18.8) | 8 (19.5) | 4 (80) | 1 (50) |
| Study design | Cross sectional | 130(96.3) | 47 (97.9) | 41 (100) | 4 (80) | 2 (100) |
|  | Unspecified | 5 (3.7) | 1 (2.1) | - | 1 (20) | - |
| Data type/source | Active survey | 123(91.1) | 44 (91.7) | 40 (97.6) | 4 (80) | 2 (100) |
|  | Retrospective | 7 (5.2) | 3 (6.3) | 1 (2.4) | - | - |
|  | Unspecified | 5 (3.7) | 1 (2.1) | - | 1 (20) | - |
| Diagnostic method used | Coprology | 1 (0.7) | 1 (2.1) | 1 (2.4) | - | - |
|  | Postmortem | 131 (97) | 46 (95.8) | 39 (95.1) | 3 (60) | 1 (50) |
|  | Molecular | 2 (1.5) | 1 (2.1) | - | 1 (20) | - |
|  | Parasitology + molecular | 1 (0.7) | - | 1 (2.4) | 1 (20) | 1 (50) |
| Parasite type* | *Echinicoccus* cyst | 79 (58.52) | 26 (50) | 21 (46.3) | 5(100) | 2 (100) |
|  | *T. saginata* (*C. bovis*) | 55 (40.74) | - | - | - | - |
|  | *T. hydatigena* | - | 14 (25) | 14 (29.3) | - | - |
|  | *T. multiceps* | - | 5 (10.4) | 3 (7.3) | - | - |
|  | *T. ovis* | - | 4 (8.3) | 4 (9.8) | - | - |
|  | Taenia egg | 1 (0.74) | 1 (2.1) | 1 (2.4) | - | - |

**References (the remaining references can be found in the reference list of the main text)**

Abebe A, Beyene D, Kumsa B. Cystic echinococcosis in cattle slaughtered at Gondar ELFORA export abattoir, northwest Ethiopia. J Parasit Dis. 2014;38(4):404-9.

Abegaz S, Mohammode A. Crossectional study on the prevalence and economic significance of hydatidosis in slaughtered ruminants at Debrezeit ELFORA export abattoir Oromia region, Eastern Showa zone, Ethiopia. Biomed J Sci Tech Res. 2018;3(3):3273-82.

Abera A, Teklebran T. Study on prevalence and cyst characterization of hydatidosis in cattle slaughtered at Wolayta Soddo municipal abbattior. Int J Res GRANTHAALAYAH. 2017;5(7):60-78.

Abiyot J, Abunna F. Prevalence of hydatidosis in small ruminants and its economic significance in Modjo modern export abattoir, Ethiopia. J Public Health Epidemiol. 2011;3(10):454-61.

Abunna F, Fentaye S, Megersa B, Regassa A. Prevalence of bovine hydatidosis in Kombolcha ELFORA abattoir, North Eastern Ethiopia. Open J Anim Sci 2012;2:281-6.

Adane M, Guadu T. Bovine hydatidosis: occurrence, economic and public health importance in Gondar ELFORA abattoir. Eur J Appl Sci. 2014;6(2):11-9.

Agegn M, Tegegne B, Tibebu S. Major causes of organ and carcass condemnation in cattle and sheep slaughtered at Bahir-Dar municipal abattoir, Amhara Regional State, Ethiopia. Advan Biol Res. 2016;10(5):323-34.

Akeberegn D, Alemneh T, Kassa T. The prevalence of bovine hydatidosis among slaughtered cattle at Debre Berhan municipal abattoir, North Shewa zone, Ethiopia. J Vet Sci Med 2017;5(1).

Alembrhan A, Haylegebriel T. Major causes of organ condemnation and economic loss in cattle slaughtered at Adigrat municipal abattoir, northern Ethiopia. Vet World. 2013;6(10):734-8.

Alemu A, Deneke Y, Ibrahim N. Major causes of organ condemnation and its financial loss in cattle in Gondar ELFORA abattoir, Ethiopia. Researcher. 2017;9(10):31-8.

Anteneh M, Asrat M, Melkamu S. Prevalence and economic significance of hydatidosis in cattle slaughter at Debretabore abattoir, North Gondar, Amhara region, Ethiopia. J Anim Res. 2015;5(3):473.

Asfaw A, Afera B. Prevalence of hydatid cyst in cattle at municipal abattoir of Shire. J Vet Sci Technol. 2014;5(3):186.

Ayele A, Gezaw E, Birhan M. Prevalence and associated risk factors of cystic echinococcosis in pigs slaughtered at Addis Ababa abattoir enterprise. Online J Anim Feed Res. 2019;9(6):225-32.

Bayew K, Ewnetu L. Prevalence and economic significance of bovine hydatidosis. Int J Agric Agrib. 2019;2(2):1-6.

Bayou K, Tolera W. Study on prevalence and economic significance of bovine hydatidosis in Woliso municipal abattoir, West Shoa, Ethiopia. Afri J Basic Appl Sci. 2016;8(5):293-8.

Bekele J, Butako B. Occurrence and financial loss assessment of cystic echinococcosis (hydatidosis) in cattle slaughtered at Wolayita Sodo municipal abattoir, Southern Ethiopia. Trop Anim Health Prod. 2011;43(1):221-8.

Belina T, Alemayehu A, Moje N, Yechale A, Girma S. Prevalence and public health significance of ovine hydatidosis in Bahir Dar town, Ethiopia. J Vet Med Anim Health. 2012;4(8):110-5.

Berhe G. Abattoir survey on cattle hydatidosis in Tigray region of Ethiopia. Tropi Anim Health Prod. 2009;41(7):1347-52.

Berihu H, Toffik K. Study on prevalence and economic significance of bovine hydatidosis in Bako municipal abattoir, West Shoa zone, Oromiya regional state. J Vet Sci Technol. 2014;5(5).

Bizuwork A, Kebede N, Tibat T, Tilahun G, Kassa T. Occurrences and financial significance of bovine cystic echinococcosis in Southern Wollo, Northeastern Ethiopia. J Vet Med Anim Health. 2013;5(2):51-6.

Brhane A, Abebed B. Epidemiological investigation of hepato-pulmonary bovine hydatidosis and its economic and zoonotic importance at Jimma municipal abattoir, Ethiopia. J Biol Agri Healthcare. 2015;5(11).

Bulcha H, Abera Z, Disassa H. Major parasitic causes of organ condemnation in bovine and its economic importance at Gimbi municipal abattoir, West Wollega zone, Ethiopia. Eur J Appl Sci. 2014;6(2):20-9.

Dana D. Prevalence and economic significance of hydatidosis in bovine slaughtered at Kindo Koysha woreda municipality abattoir, Ethiopia. Int J Res Stud Biosci. 2018;6(7):31-7.

Demissie G, Kemal J. Bovine hydatidosis and its economic importance at Kara-Alo abattoir PLC, Addis Ababa, Ethiopia. Vet Sci Technol. 2014;5(5):1.

Desta Y, Tefera M, Bekele M. Prevalence of hydatidosis of sheep slaughtered at Abergelle export abattoir, Mekelle, northern Ethiopia. Glob Vet. 2012;9(4):490-6.

Efrem L, Serda B, Sibhat B, Hirpa E. Causes of organ condemnation, its public health and financial significance in Nekemte municipal abattoir, Wollega, Western Ethiopia. J Vet Med Anim Health. 2015;7(6):205-14.

Erbeto K, Zewde G, Kumsa B. Hydatidosis of sheep and goats slaughtered at Addis Ababa abattoir: prevalence and risk factors. Trop Anim Health Prod. 2010;42(5):803-5.

Gebeyehu E. A study on prevalence and economic significance of bovine hydatidosis in Debire Birhan municipal abattoir, Centeral Ethiopia [DVM Thesis]: University of Gondar; 2015.

Gebremichael D, Feleke A, Terefe G, Lakew M. Infection rates, risk factors and cyst fertility of hydatid disease in camels in Ayssaita district, Northeastern Ethiopia. Glob Vet. 2013;11(4):465-71.

Getachew D, Almaw G, Terefe G. Occurrence and fertility rates of hydatid cysts in sheep and goats slaughtered at Modjo Luna export slaughter house, Ethiopia. Ethiop Vet J. 2012;16(1):83-91.

Getaw A, Beyene D, Ayana D, Megersa B, Abunna F. Hydatidosis: prevalence and its economic importance in ruminants slaughtered at Adama municipal abattoir, central Oromia, Ethiopia. Acta Trop. 2010;113(3):221-5.

Giro B, Hailu Y, Tilahun G, Ashenafi H. Comparative prevalence of hydatidosis in slaughtered domestic ruminants at four abattoirs of central Oromia, Ethiopia. Ethiop Vet J. 2014;18(1):29-41.

Guadu T, Gebremicael Y, Chanie M. Economic and zoonotic importance of bovine hydatidosis in Shire municipal abattoir, north west zone, Tigary region, Ethiopia. Acta Parasitol Glob. 2013;4(3):92-8.

Guduro GG, Desta AH. Cyst viability and economic significance of hydatidosis in Southern Ethiopia. J Parasitol Res. 2019;2:1-7.

Kebede N, Gebre‐Egziabher Z, Tilahun G, Wossene A. Prevalence and financial effects of hydatidosis in cattle slaughtered in Birre ‐ Sheleko and Dangila abattoirs, Northwestern Ethiopia. Zoonoses Public Health. 2011;58(1):41-6.

Kebede N, Mekonnen H, Wossene A, Tilahun G. Hydatidosis of slaughtered cattle in Wolaita Sodo abattoir, southern Ethiopia. Trop Anim Health Prod. 2009;41(4):629-33.

Kebede N. A retrospective survey of bovine hydatidosis in three abattoirs of Amhara National Regional State, northwestern Ethiopia. Trop Anim Health Prod. 2010;42(3):323-5.

Kibebew K, Bedaso A, Kuma A. Prevalence of bovine hydatidosis and its economic importance at Assela municipal abattoir. J Biol Agri Healthcare. 2016;6(23).

Kumsa B, Mohammedzein A. Prevalence, organ distribution, risk factors, and financial losses of hydatid cysts in sheep and goats slaughtered in restaurants in Jimma, South western Oromia. Comp Clin Path. 2014;23(2):333-9.

Kumsa B. Cystic echinococcosis in slaughtered cattle at Addis Ababa abattoir enterprise, Ethiopia. Vet Anim Sci. 2019;7:100050.

Lemma B, Abera T, Urga B, Niguse A, Agonafir A. Prevalence of bovine hydatidosis and its economic significance in Harar municipality abattoir, eastern Ethiopia. Am Eurasian J Sci Res. 2014;9(5):143-9.

Mekuriyaw A, Kebede G, Zenebe T, Kabeta T, Demssie A. Prevalence of bovine hydatidosis and its cyst characterization in Debre Zeit ELFORA export abattoir, Oromia Regional State, Ethiopia. Nat Sci. 2016;14(9):87-93.

Melaku A, Lukas B, Bogale B. Cyst viability, organ distribution and financial losses due to hydatidosis in cattle slaughtered at Dessie Municipal abattoir, North-Eastern Ethiopia. Vet World. 2012;5(4):213-8.

Mesele G, Guadu T, Bogale B, Chanie M. Pathological conditions causing organ and carcass condemnation and their financial losses in cattle slaughtered in Gondar, Northwest Ethiopia. Afr J Basic Appl Sci. 2015;4(6):200-8.

Moje N, Abdeta D, Kebede S, Terfa T, Desissa F, Regassa A. Major causes of organs and carcass condemnation in cattle slaughtered at Nekemte municipality abattoir, East Wollega, Ethiopia. Glob Vet. 2014;13(3):278-84.

Moje N, Degefa A. Prevalence, cyst viability, organ distributions and financial losses due to hydatidosis in cattle slaughtered at Nekemte municipal abattoir, Western Ethiopia. J Vet Med Anim Health. 2014;6(11):280-8.

Mulatu M, Mekonnen B, Tassew H, Kumar A. Bovine hydatidosis in eastern part of Ethiopia. Momona Ethiop J Sci. 2013;5(1):107-14.

Mummed YY, Webb EC. Causes of beef carcass and organ condemnations in Ethiopia. Asian J Anim Vet Adv. 2015;10(4):147-60.

Mummed YY. Beef carcass quality, yield and causes of condemnations in Ethiopia [PhD Thesis]: University of Pretoria; 2015.

Negash K, Beyene D, Kumsa B. Cystic echinococcosis in cattle slaughtered at Shashemanne municipal abattoir, South central Oromia, Ethiopia: prevalence, cyst distribution and fertility. Trans Royal Soci Trop Med Hyg. 2013;107(4):229-34.

Sheferaw D, Abdu K. Major causes of organ and carcass condemnation and associated financial losses in cattle slaughtered at Kombolcha ELFORA Abattoir from 2008-2012, Ethiopia. Ethiop Vet J. 2017;21(1):54-66.

Tadesse B, Birhanu T, Sultan A, Ayele G, Ejeta E. Prevalence, public significance and financial loss of hydatid cyst on cattle slaughtered at Nekemte municipal abattoir, Western Ethiopia. Acta Parasitol Glob. 2014;5:151-9.

Tadesse M, Tesfaye S, Admasu P. Prevalence of bovine hydatidosis and its economic importance in cattle slaughtered at Bahir Dar municipal abattoir, Northern Ethiopia. Int J Livest Res. 2018;6:1-10.

Taha B, Hassen A. The prevalence of cystic echinococcosis in cattle slaughtered in Sebeta municipal abattoir, central Ethiopia. Biomed J Sci Tech Res. 2018;6(1):4955-9.

Tefera M, Shimelis S. Prevalence and financial loss due to bovine hydatidosis at municipality abattoir of Jimma, Ethiopia. Acta Parasitol Glob. 2017;8(1):33-8.

Teshome T, Zeryehun T, Kaba T. Cystic echinococcosis: prevalence and economic significance in small ruminants slaughtered at ELFORA export abattoir, Bishoftu, Ethiopia. Ethiop Vet J. 2017;21(2):11-27.

Tilahun A, Terefe Y. Hydatidosis: prevalence, cyst distribution and economic significance in cattle slaughtered at Arbaminch municipality abattoir, Southern Ethiopia. Glob Vet. 2013;11(3):329-34.

Yohannes G, Masresha S. Study on the prevalence and associated risk factors of bovine hydatidosis in Hawassa municipal abattoir, Hawassa, Ethiopia. Gen Surg. 2019;1(3):1012.
