# Supplementary material for: A systematic review and meta-analysis on prevalence and distribution of Taenia and Echinococcus infections in Ethiopia
Source: Parasit Vectors. 2021 Sep 6;14:447. doi: 10.1186/s13071-021-04925-w (PMC8419976; doi:10.1186/s13071-021-04925-w)
Supplement: Supplementary file 4 — Additional file 4: Table S4. Characteristics of studies included in the systematic review and meta-analysis (study subject: cattle). F, female; M, male; B = both male and female; CS, cross sectional; p, prevalence; CI, confidence interval. [file 13071_2021_4925_MOESM4_ESM.doc]

| **Reference** | **Study area** | **region** | **year of study** | | **sex** | **Age (yrs)** | **Study design** | **dx method** | **Sample size** | **no +** | **P (%)** | **95% CI** | **Parasite/ disease category** |
| --- | --- | --- | --- | --- | --- | --- | --- | --- | --- | --- | --- | --- | --- |
| Abay and Kumar, 2013 | Mekelle MA | Tigray | 11, 2007 | 02, 2008 | B | mixed | CS | parasitological | 1023 | 74 | 7.23 | 5.72 - 9 | *C .bovis* |
| Abebe et al., 2014 | Gondar Elfora export Abattoir | Amhara | 11, 2010 | 04, 2011 | - | mixed | CS | parasitological | 308 | 63 | 20.5 | 16.09 -  25.4 | CE |
| Abegaz and Mohammode, 2018 | Elfora export abattoir, Debre Zeit | Oromia | 11, 2011 | 03, 2012 | - | - | CS | parasitological | 203 | 87 | 42.87 | 35.95 -  49.97 | CE |
| Abera and Teklebran, 2017 | Wolayta Soddo MA | SNNP | 11, 2012 | 03, 2013 | - | mixed | CS | parasitological | 446 | 50 | 11.21 | 8.44 -  14.51 | CE |
| Abunna et al., 2008 | hawasa MA | SNNP | 10, 2005 | 4, 2006 | B | mixed | CS | parasitological | 400 | 105 | 26.25 | 22 - 30.85 | *C .bovis* |
| Abunna et al., 2012 * | Kombolcha ELFORA abattoir | Amhara | - | - | B | mixed | CS | parasitological | 400 | 68 | 17 | 13.45 -  21.05 | CE |
| Abunna et al., 2012 * | Kombolcha ELFORA abattoir | Amhara | - | - | B | mixed | CS, retrospective | parasitological | 10068 | 1955 | 12.17 | 1865 -  20.2 | CE |
| Abunna, 2013 | Yirgalem | SNNP | 11, 2009 | 03, 2011 | B | mixed | CS | parasitological | 400 | 48 | 12 | 8.98 -  15.59 | *C .bovis* |
| Adane and Guadu, 2014 | Gondar ELFORA Abattoir | Amhara | 10, 2013 | 04, 2014 | - | mixed | CS | parasitological | 350 | 100 | 28.6 | 23.89 -  33.61 | CE |
| Adem and Alemneh, 2016* | Gondar ELFORA abattoir | Amhara | 10, 2009 | 03, 2010 | - | - | CS | parasitological | 450 | 9 | 2 | 0.92 -  3.76 | *C .bovis* |
| Agegn et al., 2016 | Bahir-Dar MA | Amhara | 10, 2015 | 03, 2016 | M | mixed | CS | parasitological | 236 | 29 | 12.3 | 8.39 -  17.17 | CE |
| Akeberegn et al., 2017 | Debreberhan MA | Amhara | - | - | B | mixed | CS | parasitological | 384 | 25 | 6.51 | 4.26 -  9.46 | CE |
| Alemu et al., 2017 | Gondar ELFORA abattoir | Amhara | 11, 2015 | 04, 2016 | B | mixed | CS | parasitological | 519 | - | - |  | CE, *C .bovis* |
| Asfaw and Afera, 2014 | Shire MA | Tigray | 05, 2014 | 06, 2014 | B | mixed | CS | parasitological | 440 | 141 | 32 | 27.7 -  36.63 | CE |
| Assefa and Tesfay, 2013 | Adigrat MA | Tigray | 10, 2012 | 04, 2013 | - | - | CS | parasitological | 745 | - | - |  | CE *C .bovis* |
| Bayew and Ewnetu, 2019 | Janamora Wereda, North Gondar | Amhara | 01, 2017 | 10, 2017 | - | - | CS | parasitological | 441 | 122 | 27.7 | 23.54 -  32.09 | CE |
| Bayou and Taddesse, 2018 | Dale Wabera District MA, Western Ethiopia | Oromia | 06, 2016 | 10, 2016 | B | - | CS | parasitological | 384 | 25 | 6.5 | 4.26 -  9.46 | *C .bovis* |
| Bayou and Tolera, 2016 | Woliso MA | Oromia | 06, 2015 | 12, 2015 | - | mixed | CS | parasitological | 390 | 107 | 27.4 | 23.07 -  32.15 | CE |
| Bedu et al., 2011 | Zeway MA | Oromia | 11, 2010 | 03, 2011 | B | - | CS | parasitological | 400 | 12 | 3 | 1.56 -  5.18 | *C .bovis* |
| Bekele and Butako, 2011 | Wolayita Sodo MA | SNNP | 11, 2009 | 04, 2010 | B | mixed | CS | parasitological | 546 | 92 | 16.85 | 13.8 -  20.26 | CE |
| Bekele et al., 2017 | Ambo MA | Oromia | ? | ? | B | mixed | CS | parasitological | 600 | 93 | 15.5 | 12.7 -  18.65 | *C .bovis* |
| Belay and Afera, 2014 | Shire MA | Tigray | 05, 2014 | 06, 2014 | B | mixed | CS | parasitological | 439 | 23 | 5.2 | 3.35 -  7.76 | *C .bovis* |
| Berhanu, 2017 | Nekemte MA | Oromia | 10, 2012 | 04, 2013 | B | mixed | CS | parasitological | 600 | 93 | 15.5 | 12.7 -  18.65 | *C .bovis* |
| Berhe, 2009 | Mekelle MA | Tigray | 11, 2006 | 10, 2007 | B | mixed | CS | parasitological | 4481 | 1439 | 32.11 | 30.75 -  33.5 | CE |
| Beyene and Hiko, 2019* | Yabello MA | Oromia | 12, 2017 | 03, 2018 | - | mixed | CS | parasitological | 384 | 128 | 33.3 |  | CE |
| Beyene and Hiko, 2019 * | Yabello MA | Oromia | 12, 2017 | 03, 2018 | - | mixed | CS | parasitological | 384 | 33 | 8.6 | 5.99 -  11.86 | *C .bovis* |
| Birhanu and Abda, 2014* | Adama MA | Oromia | 01, 2013 | 05, 2013 | B | mixed | CS | parasitological | 422 | 83 | 19.7 | 15.98 -  23.79 | *C .bovis* |
| Birhanu and Abda, 2014* | Adama MA | Oromia | 01, 2013 | 05, 2013 | B | mixed | CS | parasitological | 422 | 116 | 27.5 | 23.28 -  32.01 | CE |
| Birhanu and Abda, 2014* | Adama MA | Oromia | 01, 2013 | 05, 2013 | B | mixed | CS, retrospective | parasitological | 27180 | 2053 | 7.55 | 7.24 -  7.87 | *C .bovis* |
| Birhanu and Abda, 2014* | Adama MA | Oromia | 01, 2013 | 05, 2013 | B | mixed | CS, retrospective | parasitological | 27180 | 8735 | 32.14 | 31.58 -  32.7 | CE |
| Biruk, 2017 | Jijiga MA | Somali | 11, 2008 | 04, 2009 | B | mixed | CS | parasitological | 400 | 9 | 2.25 | 1.03 -  4.23 | *C .bovis* |
| Bizuwork et al., 2013 | Kombolcha ELFORA Industrial Abattoir | Amhara | 08, 2010 | 05, 2011 | - | - | CS | parasitological | 535 | 93 | 17.4 | 14.27 -  20.87 | CE |
| Brhane and Abebed, 2015 | Jimma MA | Oromia | 11, 2013 | 03, 2014 | B | mixed | CS | parasitological | 384 | 118 | 30.7 | 26.15 -  35.61 | CE |
| Bulcha et al., 2014 | Gimbi MA | Oromia | 06, 2013 | 09, 2014 | B | mixed | CS | parasitological | 384 | 63 | 16.4 |  | CE |
| Cheru and Zerihun, 2017 | Gondar ELFORA abattoir | Amhara | 10, 2014 | 03, 2015 | B | mixed | CS | parasitological | 400 | 22 | 5.5 | 3.48 -  8.21 | *C .bovis* |
| Dana, 2018 | Bele MA, Wolaita | SNNP | 12, 2016 | 08, 2017 | B | mixed | CS | parasitological | 384 | 56 | 14.57 | 11.21 -  18.52 | CE |
| Dawit et al., 2013 | Mekelle MA | Tigray | - | - | - | mixed | CS | parasitological | 840 | 236 | 28.09 | 25.08 -  31.27 | CE |
| Degefu and Damet, 2013 | South Wollo | Amhara | 11, 2007 | 02, 2008 | M | - | CS | parasitological | 312 | 56 | 17.95 | 13.85 -  22.67 | CE |
| Demissie and Kemal, 2014 | at Kara-Alo Abattoir PLC, AA | Addis Ababa | 11, 2013 | 04, 2014 | M | mixed | CS | parasitological | 768 | 197 | 25.7 | 22.6 -  28.89 | CE |
| Deressa et al., 2012 * | AA and Modjo abattoirs | AA, Oro | 11, 2009 | 03, 2010 | B | mixed | CS | parasitological | 61000 | 2257 | 3.7 | 3.55 -  3.85 | *C .bovis* |
| Deressa et al., 2012 * | AA and Modjo abattoirs | AA, Oro | 11, 2009 | 03, 2010 | B | mixed | CS, retrospective | parasitological | 33289 | 348 | 1.04 | 0.94 -  1.16 | *C .bovis* |
| Disassa et al., 2015 * | Dire Dawa MA | Dire Dawa | 11, 2014 | 03, 2015 | - | - | CS | parasitological | 6441 | 1460 | 22.67 |  | CE |
| Disassa et al., 2015 * | Dire Dawa MA | Dire Dawa | 11, 2014 | 03, 2015 | - | - | CS | parasitological | 6441 | 208 | 3.23 |  | *C .bovis* |
| Edao et al., 2016 | Asella Town, Tiyoworeda | Oromia | 11, 2014 | 04, 2015 | B | mixed | CS | parasitological | 430 | 5 | 1.2 | 0.38 -  2.69 | *C .bovis* |
| Korso and Edao, 2019 | Meki (East Shoa) | Oromia | 04, 2016 | 08, 2016 | B | mixed | CS | parasitological | 876 | 269 | 30.7 | 27.67 -  33.88 | *C .bovis* |
| Efrem et al., 2015 | Nekemte MA | Oromia | 10, 2013 | 04, 2014 | B | mixed | CS | parasitological | 534 | - | - |  | CE *C .bovis* |
| Emiru et al., 2015 | Elfora abattoir, Bishoftu | Oromia | 11, 2013 | 04, 2014 | B | mixed | CS | parasitological | 430 | 24 | 5.6 | 3.61 -  8.19 | *C .bovis* |
| Endris and Negussie, 2011 | Kombolcha ELFORA meat factory | Amhara | 10, 2009 | 03, 2010 | B | mixed | CS | parasitological | 420 | 28 | 6.7 | 4.48 -  9.49 | *C .bovis* |
| Engdaw et al., 2015 | Kombolcha Elfora | Amhara | 11, 2009 | 04, 2010 | B | mixed | CS | parasitological | 421 | 27 | 6.4 | 4.27 -  9.19 | *C .bovis* |
| Firew and Moges, 2014 | Jimma | Oromia | 11, 2013 | 04, 2014 | B | mixed | CS | parasitological | 547 | 28 | 5.1 | 3.43 -  7.31 | *C .bovis* |
| Gebeyehu, 2015 | Debre Berhan | Amhara | 11, 2014 | 04, 2015 | B | mixed | CS | parasitological | 384 | 110 | 28.6 | 24.17 -  33.45 | CE |
| Geinoro and Bedore, 2019 | Bishoftu MA | Oromia | 10, 2013 | 04, 2014 | B | mixed | CS | parasitological | 371 | 29 | 7.8 | 5.3 -  11.03 | *C .bovis* |
| Getachew et al., 2017 * | Mekelle MA | Tigray | 11, 2009 | 03, 2010 | - | mixed | CS | parasitological | 1800 | 399 | 22.2 | 20.27 -  24.16 | CE |
| Getachew et al., 2017* | Mekelle MA | Tigray | 11, 2009 | 03, 2010 | - | mixed | CS | parasitological | 1800 | 80 | 4.44 | 3.54 -  5.5 | *C .bovis* |
| Getaw et al., 2010 * | Adama MA | Oromia | 11, 2007 | 04, 2008 | B | mixed | CS | parasitological | 852 | 399 | 46.8 | 43.44 -  50.25 | CE |
| Getaw et al., 2010 * | Adama MA | Oromia | 11, 2007 | 04, 2008 | B | mixed | CS, retrospective | parasitological | 107333 | 26080 | 24.3 |  | CE |
| Giro et al., 2014 | central Oromia | Oromia | 10, 2010 | 05, 2012 | - | - | CS | parasitological | 2910 | 1896 | 65.15 | 63.39 -  66.89 | CE |
| Guadu et al., 2013 | Shire MA | Tigray | 10, 2010 | 12, 2010 | - | - | CS | parasitological | 540 | 140 | 25.92 | 22.28 -  29.84 | CE |
| Guduro and Desta, 2019 | Hawassa MA | SNNP | 11, 2016 | 04, 2017 | B | mixed | CS | parasitological | 400 | 208 | 52 | 46.98 -  56.99 | CE |
| Berihu and Toffik, 2014 | Bako MA, West Shoa | Oromia | 11, 2011 | 04, 2012 | B | mixed | CS | parasitological | 246 | 29 | 11.88 | 8.04 -  16.49 | CE |
| Hailemariam et al., 2014 | Haramaya,Dire Dawa, Jijiga and Addis Ababa | Oro, DD, Som, AA | 2010 | 2012 | - | - | CS | molecular | - | 41 | - |  | *C .bovis* |
| Hailemariam et al., 2012* | Aweday, Jigjiga, haramaya, AA abattoirs | Somali, oromia, AA | 06, 2010 | 02, 2011 | - | - | - | molecular | - | 16 |  |  | CE |
| Hiko et al., 2018 | Bishoftu | Oromia | - | - | - | - | - | parasitological | 662 | 335 | 51 | 46.72 -  54.48 | CE |
| Hirpha et al., 2016 | Halaba Kulito, South Ethi | SNNP | 12, 2015 | 04, 2016 | B | mixed | CS | parasitological | 384 | 33 | 8.6 | 5.99 -  11.86 | *C .bovis* |
| Ibrahim, 2000 | Nekemte MA | Oromia | 10, 1987 | 03, 1990 | B | mixed | CS | parasitological | 1355 | 286 | 21 | 18.96 -  23.38 | *C .bovis* |
| Ibrahim and Zerihun, 2012 | AA abatoir | Addis Ababa | 10, 2010 | 03, 2011 | B | mixed | CS | parasitological | 535 | 19 | 3.6 | 2.15 -  5.49 | *C .bovis* |
| Jones et al., 2012 | Wondo Genet | SNNP | - | - | B | mixed | CS | parasitological | 320 | 151 | 47.2 | 41.61 -  52.82 | CE |
| Kassaw et al., 2017 | Kombolcha ELFORA Meat Processing factory | Amhara | 10, 2015 | 04, 2016 | B | mixed | CS | parasitological | 425 | 20 | 4.7 | 2.9 - 7.17 | *C .bovis* |
| Kebede, 2010 | Finote Selam, Injibara, and Gondar | Amhara | - | - | B | mixed | CS, retrospective | parasitological | 22755 | 12594 | 55.35 | 54.7 -  55.99 | CE |
| Kebede et al., 2009 * | AA abatoir | Addis Ababa | 09, 2004 | 08, 2005 | B | mixed | CS | parasitological | 11227 | 842 | 7.5 | 7.02 - 8 | CE |
| Kebede, 2008 | Amhara | Amhara | 09, 2005 | 02, 2007 | B | mixed | CS | parasitological | 4456 | 824 | 18.49 | 17.36 -  19.66 | *C .bovis* |
| Kebede et al., 2008 | AA abatoir | Addis Ababa | - | - | B | mixed | CS | parasitological | 522 | 39 | 7.5 | 5.37 -  10.07 | *C .bovis* |
| Kebede et al., 2009 * | Tigray | Tigray | - | - | - | - | CS | parasitological | 5194 | 1146 | 22.1 | 20.94 -  23.22 | CE |
| Kebede et al., 2009 * | Debre Markos MA | Amhara | 09, 2007 | 08, 2008 | B | mixed | CS | parasitological | 413 | 202 | 48.9 | 43.99 -  53.85 | CE |
| Kebede et al., 2009* | Wolaita Sodo abattoir | SNNP | 07, 2007 | 06, 2008 | - | - | CS | parasitological | 400 | 64 | 16 | 12.55 -  19.97 | CE |
| Kebede et al., 2011 | Birre-Sheleko and Dangila Abattoirs | Amhara | 08, 2007 | 07, 2008 | B | mixed | CS | parasitological | 521 | 79 | 15.2 | 12.19 -  18.54 | CE |
| Kibebew, 2016 | Assela MA | Oromia | 10, 2012 | 03, 2013 | - | mixed | CS | parasitological | 384 | 170 | 44.27 | 39.23 -  49.4 | CE |
| Kinfe et al., 2016 | Gondar Elfora | Amhara | 10, 2015 | 04, 2016 | - | mixed | CS | parasitological | 400 | 9 | 2.25 | 1.03 -  4.23 | *C .bovis* |
| Kumsa, 2019 | AA Abattoir enterprise | Addis Ababa | 10, 2015 | 05, 2016 | B | mixed | CS | parasitological | 1209 | 254 | 21 | 18.74 -  23.42 | CE |
| Lemma et al., 2014 | Harar MA | Harar | 12, 2013 | 03, 2014 | B | mixed | CS | parasitological | 679 | 77 | 11.3 | 22.44 -  33.24 | CE |
| Mame and Amante, 2019* | Enango MA, Western Ethiopia | Oromia | 04, 2017 | 11, 2017 | - | mixed | CS | parasitological | 400 | 59 | 14.75 |  | CE |
| Mame and Amante, 2019* | Enango MA, Western Ethiopia | Oromia | 04, 2017 | 11, 2017 | - | mixed | CS | parasitological | 400 | 30 | 7.5 |  | *C .bovis* |
| Megersa et al., 2010 | Jimma MA | Oromia | 11, 2008 | 03, 2009 | - | - | CS | parasitological | 500 | 22 | 4.4 | 2.78 -  6.59 | *C .bovis* |
| Mekonnen, 2017 | Kofale District MA | Oromia | 09, 2015 | 04, 2016 | B | mixed | CS | parasitological | 768 | 38 | 4.94 | 3.52 -  6.73 | *C .bovis* |
| Mekuriyaw et al., 2016 | Debre Zeit Elfora Export Abattoir | Oromia | 12, 2015 | 03, 2016 | - | mixed | CS | parasitological | 384 | 69 | 17.97 | 14.26 -  22.18 | CE |
| Melaku et al., 2012 | Dessie MA | Amhara | 10, 2010 | 03, 2011 | - | mixed | CS | parasitological | 610 | 83 | 13.61 | 10.99 -  16.59 | CE |
| Mersie, 1993 | Assebe Teferi | Oromia | - | - | B | mixed | CS | parasitological | 171 | 35 | 20.5 | 14.69 -  27.3 | CE |
| Mesele et al., 2012 | Gondar ELFORA abattoir | Amhara | 11, 2011 | 04, 2012 | M | mixed | CS | pathological | 1550 | 268 | 17.29 |  | CE |
| Mohammed et al., 2012 * | Kombolcha Elfora Abattoir | Amhara | 11, 2011 | 04, 2012 | B | mixed | CS | parasitological | 1175 | 168 | 14.32 |  | CE |
| Mohammed et al., 2012 * | Kombolcha Elfora Abattoir | Amhara | 11, 2012 | 04, 2013 | B | mixed | CS | parasitological | 1175 | 63 | 5.37 |  | *C .bovis* |
| Moje et al., 2014 * | Nekemte MA | Oromia | 12, 2012 | 06, 2013 | - | mixed | CS | parasitological | 433 | 115 | 26.55 | 22.46 -  30.99 | CE |
| Moje et al., 2014 * | Shashemene MA | Oromia | 12, 2011 | 05, 2012 | B | mixed | CS | parasitological | 405 | 41 | 10.1 | 7.36 -  13.48 | *C .bovis* |
| Moje et al., 2014 * | Shashemene MA | Oromia | 12, 2011 | 05, 2012 | B | mixed | CS | parasitological | 405 | 203 | 50.1 | 45.14 -  55.1 | CE |
| Moje et al., 2014 * | Shashemene MA | Oromia | 12, 2011 | 05, 2012 | B | mixed | CS, retrospective | parasitological | 17187 | 1955 | 11.37 | 10.9 -  11.86 | *C .bovis* |
| Moje et al., 2014 * | Shashemene MA | Oromia | 12, 2011 | 05, 2012 | B | mixed | CS, retrospective | parasitological | 17187 | 8654 | 50.35 | 49.6 -  51.1 | CE |
| Moje and Degefa, 2014 | Nekemte MA | Oromia | 10, 2013 | 03, 2014 | B | mixed | CS | parasitological | 473 | 82 | 17.34 | 14.03 -  21.05 | CE |
| Mulatu et al., 2013 | Dire Dawa MA | Dire Dawa | 11, 2010 | 03, 2011 | ? | mixed | CS | parasitological | 1536 | 308 | 20.05 | 18.08 -  22.14 | CE |
| Mulugeta et al., 2015 | Debretabore Abattoir | Amhara | 07, 2012 | 09, 2012 | B | - | CS | parasitological | 384 | 106 | 27.64 | 23.19 -  32.37 | CE |
| Mummed, 2015 | 8 Eth export abattoirs | eth | - | - | B | mixed | CS | parasitological | 62917 | - | - |  | CE, *C .bovis* |
| Negash et al., 2013 | Shashemanne MA | Oromia | 12, 2010 | 03, 2011 | B | mixed | CS | parasitological | 384 | 190 | 49.5 | 44.37 -  54.6 | CE |
| Regassa et al., 2006 | western Oromia | Oromia | 2003 | 2004 | B | mixed | CS | parasitological | 257 | 1 | 0.4 | 0.01 -  2.15 | Taeniasis |
| Regassa et al., 2009 * | Wolaita Soddo MA | SNNP | 11, 2007 | 04, 2008 | B | mixed | CS | parasitological | 415 | 64 | 15.42 | 12.08 -  19.26 | CE |
| Regassa et al., 2009 * | Wolaita Soddo MA | SNNP | 11, 2007 | 04, 2008 | B | mixed | CS | parasitological | 415 | 47 | 11.33 | 8.44 -  14.77 | *C .bovis* |
| Regassa et al., 2010 | hawasa MA | SNNP | 12, 2008 | 03, 2009 | B | mixed | CS | parasitological | 632 | 333 | 52.69 | 48.71 -  56.64 | CE |
| Roming et al., 2011 | Ethiopia | Eth | - | - | - | - | review | parasitological | - | - | - |  | CE |
| Sheferaw and Abdu, 2017 | Kombolcha ELFORA abattoir | Amhara | 10, 2013 | 04, 2014 | - | - | CS | parasitological | 1200 | - | - |  | CE |
| Shiferaw et al., 2009 * | Mekelle MA | Tigray | - | - | - | - | - | parasitological | 1022 | 330 | 32.28 | 29.43 -  35.25 | CE |
| Shiferaw et al., 2009 * | Mekelle MA | Tigray | - | - | - | - | - | parasitological | 1022 | 75 | 7.33 | 5.82 -  9.11 | *C .bovis* |
| Tadesse et al., 2013 | Sebeta, Tulu Bolo and Weliso abattoirs | Oromia | - | - | B | mixed | CS | parasitological | 1216 | 56 | 4.6 | 3.5 - 5.94 | *C .bovis* |
| Tadesse et al., 2018 | Bahir Dar MA | Amhara | 11, 2013 | 04, 2014 | - | mixed | CS | parasitological | 384 | 72 | 18.75 | 14.97 -  23.02 | CE |
| Tadesse et al., 2014 | Nekemte MA | Oromia | 12, 2013 | 04, 2014 | B | mixed | CS | parasitological | 531 | 91 | 17.1 | 14.03 -  20.62 | CE |
| Taha and Hassen, 2018 | sebeta slaughterhouse | Oromia | 11, 2008 | 04, 2009 | - | - | CS | parasitological | 376 | 66 | 17.6 | 13.84 -  21.78 | CE |
| Tamirat et al., 2018 | Bahir Dar MA | Amhara | 11, 2016 | 04, 2017 | - | mixed | CS | parasitological | 480 | 20 | 4.2 | 2.56 -  6.36 | *C .bovis* |
| Tefera et al., 2016 | Dessie MA | Amhara | 11, 2013 | 04, 2014 | - | mixed | CS | parasitological | 768 | 170 | 22.13 | 19.25 -  25.24 | CE |
| Tefera et al., 2016 | Dessie MA | Amhara | 11, 2013 | 04, 2014 | - | mixed | CS | parasitological | 768 | 6 | 0.78 |  | *C .bovis* |
| Tefera and Shimelis, 2017 | Jimma MA | Oromia | 11, 2009 | 04, 2010 | - | mixed | CS | parasitological | 512 | 226 | 44.14 | 39.79 -  48.56 | CE |
| Tegegne et al., 2018 | Kombolcha ELFORA abattoir | Amhara | 11, 2016 | 04, 2017 | B | mixed | CS | parasitological | 234 | 21 | 8.97 | 5.64 -  13.39 | *C .bovis* |
| Terefe et al., 2014 | Harar MA | Harar | 10, 2009 | 09, 2010 | B | mixed | CS | parasitological | 898 | 177 | 19.7 | 17.16 -  22.47 | *C .bovis* |
| Terefe et al., 2019 | Harar, Dire Dawa and Haramaya | Har, DD, Oro | 02, 2015 | 09, 2016 | - | - | CS | para + molecular | 891 | 75 | 8.4 | 6.68 -  10.44 | CE |
| Terefe et al., 2012 | AA abattoir enterprise | Addis Ababa | 11, 2009 | 04, 2010 | M | mixed | CS | parasitological | 484 | 196 | 40.5 | 36.09 -  45.02 | CE |
| Tesfaye, 2016 | Debrezeit MA | Oromia | 11, 2010 | 03, 2011 | - | mixed | CS | parasitological | 700 | 38 | 5.43 | 3.87 -  7.38 | *C .bovis* |
| Tesfaye et al., 2012 | Wolaita soddo | SNNP | - | - | - | - | CS | parasitological | 540 | 14 | 2.59 | 1.42 -  4.31 | *C .bovis* |
| Tigre et al., 2016 | Jimma, AA abattoirs | Oromia | 01, 2010 | 10, 2011 | B | - | CS | para + molecular | - | 97 | - |  | CE |
| Tilahun and Terefe, 2013 | Arbaminch MA | SNNP | 10, 2009 | 04, 2010 | - | - | CS | parasitological | 600 | 123 | 20.5 | 17.34 -  23.96 | CE |
| Tolossa et al., 2009 * | Jimma MA | Oromia | 10, 2007 | 03, 2008 | M | mixed | CS | parasitological | 512 | 15 | 2.93 | 1.65 -  4.79 | *C .bovis* |
| Tolosa et al., 2009 * | Jimma MA | Oromia | 10, 2007 | 03, 2008 | M | mixed | CS | parasitological | 512 | 161 | 31.44 | 27.44 -  35.66 | CE |
| Tolossa et al., 2015 | Adama MA | Oromia | 11, 2013 | 04, 2014 | - | - | CS | parasitological | 384 | 10 | 2.6 | 1.26 -  4.74 | *C .bovis* |
| Wondimagegnei and Belete, 2015 | Debreberhan | Amhara | 11, 2010 | 03, 2011 | B | mixed | CS | parasitological | 384 | 18 | 4.64 | 2.8 - 7.31 | *C .bovis* |
| Worku, 2017 * | Bishoftu Elfora Export Abattoir | Oromia | 11, 2015 | 03, 2016 | M | mixed | CS | parasitological | 384 | 17 | 4.4 | 2.6 - 6.99 | *C .bovis* |
| Worku, 2017 * | Bishoftu Elfora Export Abattoir | Oromia | 11, 2015 | 03, 2016 | M | mixed | CS | parasitological | 384 | 47 | 12.2 | 9.13 -  15.94 | CE |
| Yalew et al., 2017 * | Bahir Dar MA | Amhara | 11, 2016 | 05, 2017 | - | mixed | CS | parasitological | 768 | 20 | 2.6 | 1.6 - 3.99 | CE |
| Yalew et al., 2017 * | Bahir Dar MA | Amhara | 11, 2017 | 05, 2018 | - | mixed | CS | parasitological | 768 | 15 | 2 | 1.1 - 3.2 | *C .bovis* |
| Mummed and Webb | Abergelle, Elfora Bishoftu and Elfora Kombolcha abattoirs | Tig, oro, Amh | 02, 2010 | 11, 2013 | - | - | CS | parasitological | 62917 | - | - |  | CE, *C. bovis* |
| Yigizaw et al., 2017 | Dessie MA | Amhara | 11, 2015 | 04, 2014 | B | mixed | CS | parasitological | 384 | 26 | 6.8 | 4.47 -  9.76 | *C. bovis* |
| Yimer and Gebrmedehan, 2019 | Debre Brihan | Amhara | 12, 2016 | 04, 2017 | B | mixed | CS | parasitological | 405 | 22 | 5.43 | 3.44 -  8.11 | *C. bovis* |
| Yohannes and Masresha, 2019 | Hawassa MA | SNNP | 11, 2018 | 04, 2019 | B | mixed | CS | parasitological | 395 | 158 | 40.2 | 35.13 -  45.02 | CE |
