# Supplementary material for: A systematic review and meta-analysis on prevalence and distribution of Taenia and Echinococcus infections in Ethiopia
Source: Parasit Vectors. 2021 Sep 6;14:447. doi: 10.1186/s13071-021-04925-w (PMC8419976; doi:10.1186/s13071-021-04925-w)
Supplement: Supplementary file 5 — Additional file 5: Table S5. Characteristics of studies included in the systematic review and meta-analysis (study subject: sheep). F, female; M, male; B = both male and female; CS, cross sectional; p, prevalence; CI, confidence interval. [file 13071_2021_4925_MOESM5_ESM.doc]

| **Reference** | **Study area** | **region** | **year of study** | | **sex** | **Age (yrs)** | **Study design** | **dx method** | **Sample size** | **no +** | **P (%)** | **95% CI LL-UL** | **Parasite/ disease category** |
| --- | --- | --- | --- | --- | --- | --- | --- | --- | --- | --- | --- | --- | --- |
| Haile, 2019* | Allana, Abysinia & Elfora export abattoirs | Oromia | 11, 2018 | 03, 2019 | - | mixed | CS | parasitological | 384 | 22 | 5.7 | 3.63 -  8.55 | *T. ovis* |
| Haile, 2019* | Allana, Abysinia & Elfora export abattoirs | Oromia | 11, 2018 | 03, 2019 | - | mixed | CS | parasitological | 384 | 64 | 16.7 | 13.08 -  20.78 | *T. hydatigena* |
| Haile, 2019* | Allana, Abysinia & Elfora export abattoirs | Oromia | 11, 2018 | 03, 2019 | - | mixed | CS | parasitological | 384 | 53 | 13.8 | 10.51 -  17.66 | CE |
| Abede and Esayas, 2001* | D/ziet export abattoir | Oromia | 09, 1998 | 03, 1999 | - | - | CS | parasitological | 92 | 30 | 32.85 | 23.20 -  43.18 | *T. hydatigena* |
| Abede and Esayas, 2001* | D/ziet export abattoir | Oromia | 09, 1998 | 03, 1999 | - | - | CS | Parasitological, copro | 92 | 9 | 9.88 | 4.57 -  17.76 | Cestode egg |
| Abegaz and Mohammode, 2018 | Elfora export abattoir, Debre Zeit | Oromia | 11, 2011 | 03, 2012 | - | - | CS | parasitological | 384 | 27 | 7.03 | 4.56 -  9.81 | CE |
| Abiyot and Abunna, 2011 * | Modjo Modern Export Abattoir | Oromia | 11, 2009 | 04, 2010 | - | - | CS | parasitological | 348 | 28 | 8.05 | 5.41 -  11.42 | CE |
| Abiyot and Abunna, 2011 * | Modjo Modern Export Abattoir | Oromia | 11, 2009 | 04, 2010 | - | - | CS, retrospective | parasitological | 457776 | 24903 | 5.44 | 5.37 -  5.51 | CE |
| Achenef et al., 1999 * | Debre Berhan | Amhara | 1996 | 1997 | B | Mixed | CS | parasitological, clinical obser + post mortem | 220 | 42 | 19.09 | 14.12 -  24.92 | *T. multiceps* |
| Achenef et al., 1999* | Debre Berhan | Amhara | 1996 | 1997 | B | Mixed | CS, retrospective | parasitological | 373 | 13 | 3.4 | 1.87 -  5.89 | *T. multiceps* |
| Admasu et al., 2019 | Bishoftu Elfora Export Abattoir | Oromia | 11, 2017 | 04, 2018 | - | mixed | CS | parasitological | 232 | 42 | 18.1 | 13.37 -  23.67 | *T. hydatigena* |
| Agegn et al., 2016 | Bahir-Dar MA | Amhara | 10, 2015 | 03, 2016 | M | mixed | CS | parasitological | 148 | 3 | 2 | 0.42 -  5.81 | CE |
| Assefa et al., 2015 | AA abatoir | Addis Ababa | 10, 2011 | 03, 2012 | B | mixed | CS | parasitological | 262 | 21 | 8.02 | 5.03 -  11.99 | CE |
| Ayele et al., 2016 * | HNELMEIA | Oromia | - | - | - | mixed | CS | parasitological | 384 | 44 | 11.46 | 8.45 -  15.08 | *T. hydatigena* |
| Ayele et al., 2016 * | HNELMEIA | Oromia | - | - | - | mixed | CS | parasitological | 384 | 13 | 3.39 | 1.81 -  5.72 | CE |
| Ayele et al., 2016 * | HNELMEIA | Oromia | - | - | - | mixed | CS | parasitological | 384 | 11 | 2.86 | 1.44 –  5.07 | *T. ovis* |
| Ayele et al., 2016 * | HNELMEIA | Oromia | - | - | - | mixed | CS | parasitological | 384 | 13 | 3.3 | 1.81 -  5.72 | *T. multiceps* |
| Bayu et al., 2013 * | AA abattoir enterprise | Addis Ababa | 12, 2011 | 04, 2012 | B | mixed | CS | parasitological | 576 | 45 | 7.81 | 5.75 -  10.31 | *T. hydatigena* |
| Bayu et al., 2013 * | AA abattoir enterprise | Addis Ababa | 12, 2011 | 04, 2012 | B | mixed | CS | parasitological | 576 | 23 | 3.99 | 2.55 -  5.93 | CE |
| Belina et al., 2012 | Bahir Dar | Amhara | 10, 2010 | 04, 2011 | B | mixed | CS | parasitological | 400 | 60 | 15 | 11.65 -  18.88 | CE |
| Birhanu, 2014 | Adama MA | Oromia | 12, 2013 | 04, 2014 | B | mixed | CS | parasitological | 384 | 231 | 60.2 | 55.07 -  65.09 | CE |
| Degefu and Damet, 2013 | South Wollo | Amhara | 11, 2007 | 02, 2008 | M | - | CS | parasitological | 197 | 15 | 7.61 | 4.32 -  12.25 | CE |
| Desta et al., 2012 | Abergelle Export Abattoir | Tigray | 11, 2010 | 03, 2011 | - | mixed | CS | parasitological | 1152 | 134 | 11.6 | 9.84 -  13.63 | CE |
| Erbeto et al., 2010 | AA abatoir | Addis Ababa | 10, 2007 | 05, 2008 | B | mixed | CS | parasitological | 1033 | 206 | 19.9 | 17.55 -  22.51 | CE |
| Gessese et al., 2014 * | Dessie MA | Amhara | 11, 2011 | 03, 2012 | B | mixed | CS | parasitological | 510 | 233 | 45.69 | 41.30 -  50.12 | *T. hydatigena* |
| Gessese et al., 2014 * | Dessie MA | Amhara | 11, 2011 | 03, 2012 | B | mixed | CS | parasitological | 510 | 43 | 8.43 | 6.17 -  11.19 | *T. ovis* |
| Gessese et al., 2014 * | Dessie MA | Amhara | 11, 2011 | 03, 2012 | B | mixed | CS | parasitological | 510 | 46 | 9.02 | 6.68 -  11.85 | CE |
| Getachew et al., 2012 | Modjo Luna Export Slaughter House | Oromia | 12, 2009 | 02, 2010 | - | mixed | CS | parasitological | 325 | 25 | 7.7 | 5.04 -  11.15 | CE |
| Getaw et al., 2010 | Adama MA | Oromia | 11, 2007 | 04, 2008 | B | mixed | CS | parasitological | 92 | 27 | 29.3 | 20.31 -  39.76 | CE |
| Giro et al., 2014 | central Oromia | Oromia | 10, 2010 | 05, 2012 | - | - | CS | parasitological | 6680 | 611 | 9.15 | 8.47 -  9.86 | CE |
| Guadu et al., 2012 | Hashim Nur’s Meat Export Abattoir | Oromia | 12, 2010 | 03, 2011 | - | mixed | CS | parasitological | 395 | 143 | 36.2 | 31.46 -  41.16 | *T. hydatigena* |
| Hailemariam et al., 2012 | Aweday, Jigjiga, haramaya, AA abattoirs | Somali, oromia, AA | 06, 2010 | 02, 2011 | - | - | - | molecular | - | 11 | - |  | CE |
| Jibat et al., 2008 | HELMEX abattoir | Oromia | 12, 2005 | 06, 2006 | B | mixed | CS | parasitological | 1152 | - | - |  | *T. hydatigena*, CE |
| Kumsa and Mohammedzein, 2014 | Jimma | Oromia | 11, 2010 | 04, 2011 | B | mixed | CS | parasitological | 502 | 149 | 29.5 | 25.72 -  33.89 | CE |
| Mandefro et al., 2015 * | Bishoftu Elfora Export Abattoir | Oromia | 10, 2013 | 04, 2014 | B | mixed | CS | parasitological | 384 | 22 | 5.73 | 3.63 -  8.55 | *T. hydatigena* |
| Mandefro et al., 2015 * | Bishoftu Elfora Export Abattoir | Oromia | 10, 2013 | 04, 2014 | B | mixed | CS | parasitological | 384 | 5 | 1.3 | 0.42 -  3.01 | CE |
| Mekuria et al., 2013 | Dire Dawa MA | Dire Dawa | - | - | B | mixed | CS | parasitological | 420 | 96 | 22.8 | 18.80 -  26.87 | *T. hydatigena* |
| Mengistu et al., 2017 | Bishoftu Elfora Export Abattoir | Oromia | 11, 2014 | 04, 2015 | B | mixed | CS | grossly, naked eye | 384 | 19 | 4.9 | 3.01 -  7.62 | *T. multiceps* |
| Regassa et al., 2006 | western Oromia | Oromia | 2003 | 2004 | B | mixed | CS | parasitological | 255 | 0 | 0 | 0.00 -  1.44 | Tapeworm infection |
| Regassa et al., 2013 | Luna Export Abattoir | Oromia | 12, 2009 | 04, 2010 | - | - | CS | parasitological | 451 | - | - |  | *T. hydatigena*, *T. ovis*, CE |
| Samuel and Zewde, 2010 | HASHIM-NUR Export Abattoir, Debre Zeit | Oromia | - | - | - | mixed | CS | parasitological | 630 | 252 | 40 | 36.15 -  43.94 | *T. hydatigena* |
| Sissay et al., 2008 * | Haramaya, Harar, Dire-Dawa and Jijiga abattoirs | Oro, Har, DD, Som | 05, 2003 | 04, 2005 | B | mixed | CS | parasitological | 655 | 168 | 26 | 22.34 -  29.17 | *T. ovis* |
| Sissay et al., 2008 * | Haramaya, Harar, Dire-Dawa and Jijiga abattoirs | Oro, Har, DD, Som | 05, 2003 | 04, 2005 | B | mixed | CS | parasitological | 655 | 518 | 79 | 75.77 -  82.14 | *T. hydatigena* |
| Sissay et al., 2008 * | Haramaya, Harar, Dire-Dawa and Jijiga abattoirs | Oro, Har, DD, Som | 05, 2003 | 04, 2005 | B | mixed | CS | parasitological | 655 | 444 | 68 | 64.06 -  71.35 | CE |
| Terefe et al., 2019 | Harar, Dire Dawa and Haramaya | Har, DD, Oro | 02, 2015 | 09, 2016 | - | - | CS | para + molecular | 95 | 1 | 1.1 | 0.03 -  5.73 | CE |
| Teshome et al., 2017 | ELFORA export abattoir | Oromia | 11, 2015 | 04, 2016 | - | mixed | CS | parasitological | 400 | 57 | 14.3 | 10.97 -  18.07 | CE |
| Wondimu et al., 2011 | Hashim Nurs’ Ethiopian livestock and Meat export abattoir | Oromia | 10, 2010 | 04, 2011 | - | mixed | CS | parasitological | 576 | 327 | 56.8 | 52.61 -  60.86 | *T. hydatigena* |
| Worku, 2017 * | Bishoftu Elfora Export Abattoir | Oromia | 11, 2015 | 03, 2016 | B | mixed | CS | parasitological | 385 | 31 | 8.1 | 5.54 -  11.23 | CE |
| Worku, 2017 * | Bishoftu Elfora Export Abattoir | Oromia | 11, 2015 | 03, 2016 | B | mixed | CS | parasitological | 385 | 10 | 2.6 | 1.25 -  4.72 | *T. multiceps* |
