# Supplementary material for: A systematic review and meta-analysis on prevalence and distribution of Taenia and Echinococcus infections in Ethiopia
Source: Parasit Vectors. 2021 Sep 6;14:447. doi: 10.1186/s13071-021-04925-w (PMC8419976; doi:10.1186/s13071-021-04925-w)
Supplement: Supplementary file 6 — Additional file 6: Table S6. Characteristics of studies included in the systematic review and meta-analysis (study subject: goat). F, female; M, male; B = both male and female; CS, cross sectional; p, prevalence; CI, confidence interval. [file 13071_2021_4925_MOESM6_ESM.doc]

| **Reference** | **Study area** | **region** | **year of study** | | **sex** | **Age (yrs)** | **Study design** | **dx method** | **Sample size** | **no +** | **P (%)** | **95% CI** | **Parasite/ disease category** |
| --- | --- | --- | --- | --- | --- | --- | --- | --- | --- | --- | --- | --- | --- |
| Haile, 2019 * | Allana, Abysinia & Elfora export abattoirs | Oromia | 11, 2018 | 03, 2019 | - | mixed | CS | parasitological | 384 | 8 | 2.1 | 0.90 - 4.06 | *T. ovis* |
| Haile, 2019* | Allana, Abysinia & Elfora export abattoirs | Oromia | 11, 2018 | 03, 2019 | - | mixed | CS | parasitological | 384 | 44 | 11.5 | 8.45 - 15.08 | *T. hydatigena* |
| Haile, 2019* | Allana, Abysinia & Elfora export abattoirs | Oromia | 11, 2018 | 03, 2019 | - | mixed | CS | parasitological | 384 | 33 | 8.6 | 5.99 -  11.86 | CE |
| Abede and Esayas, 2001* | D/ziet export abattoir | Oromia | 09, 1998 | 03, 1999 | - | - | CS | parasitological | 91 | 31 | 34 | 24.45 -  44.75 | *T. hydatigena* |
| Abede and Esayas, 2001* | D/ziet export abattoir | Oromia | 09, 1998 | 03, 1999 | - | - | CS | parasitological | 91 | 9 | 9.5 | 4.62 -  17.95 | Cestode |
| Abegaz and Mohammode, 2018 | Elfora export abattoir, Debre Zeit | Oromia | 11, 2011 | 03, 2012 | - | - | CS | parasitological | 547 | 24 | 4.39 | 2.83 -  6.46 | CE |
| Abiyot and Abunna, 2011* | Modjo Modern Export Abattoir | Oromia | 11, 2009 | 04, 2010 | - | - | CS | parasitological | 767 | 69 | 8.99 | 7.07 -  11.25 | CE |
| Abiyot and Abunna, 2011* | Modjo Modern Export Abattoir | Oromia | 11, 2009 | 04, 2010 | - | - | CS, retrospective | parasitological | 1083575 | 119085 | 10.99 | 10.93 -  11.05 | CE |
| Admasu et al., 2019 | Bishoftu Elfora Export Abattoir | Oromia | 11, 2017 | 04, 2018 | - | mixed | CS | parasitological | 268 | 60 | 22.4 | 17.54 -  27.86 | *T. hydatigena* |
| Assefa et al., 2015 | AA abattoir | Addis Ababa | 10, 2011 | 03, 2012 | B | mixed | CS | parasitological | 250 | 17 | 6.8 | 4.01 -  10.66 | CE |
| Ayele et al., 2016 * | HNELMEI abattoir | Oromia | - | - | - | mixed | CS | parasitological | 384 | 49 | 12.76 | 9.59 -  16.52 | *T. hydatigena* |
| Ayele et al., 2016 * | HNELMEI abattoir | Oromia | - | - | - | mixed | CS | parasitological | 384 | 2 | 0.52 | 0.06 - 1.87 | CE |
| Ayele et al., 2016 * | HNELMEI abattoir | Oromia | - | - | - | mixed | CS | parasitological | 384 | 14 | 3.65 | 2.01 - 6.04 | *T. ovis* |
| Ayele et al., 2016 * | HNELMEI abattoir | Oromia | - | - | - | mixed | CS | parasitological | 384 | 2 | 0.52 | 0.06 - 1.87 | *T. multiceps* |
| Bayu et al., 2013 * | AA abattoir enterprise | Addis Ababa | 12, 2011 | 04, 2012 | B | mixed | CS | parasitological | 576 | 91 | 15.8 | 12.91 -  19.04 | *T. hydatigena* |
| Bayu et al., 2013 * | AA abattoir enterprise | Addis Ababa | 12, 2011 | 04, 2012 | B | mixed | CS | parasitological | 576 | 8 | 1.39 | 0.60 - 2.72 | CE |
| Erbeto et al., 2010 | AA abatoir | Addis Ababa | 10, 2007 | 05, 2008 | B | mixed | CS | parasitological | 639 | 102 | 16 | 13.21 -  19.04 | CE |
| Gessese et al., 2014 * | Dessie MA | Amhara | 11, 2011 | 03, 2012 | B | mixed | CS | parasitological | 420 | 304 | 72.38 | 67.84 -  76.60 | *T. hydatigena* |
| Gessese et al., 2014 * | Dessie MA | Amhara | 11, 2011 | 03, 2012 | B | mixed | CS | parasitological | 420 | 36 | 8.57 | 10.23 -  16.96 | *T. ovis* |
| Gessese et al., 2014 * | Dessie MA | Amhara | 11, 2011 | 03, 2012 | B | mixed | CS | parasitological | 420 | 8 | 1.9 | 0.83 - 3.72 | CE |
| Getachew et al., 2012 | Modjo Luna Export Slaughter House | Oromia | 12, 2009 | 02, 2010 | - | mixed | CS | parasitological | 440 | 27 | 6.13 | 4.08 - 8.80 | CE |
| Getaw et al., 2010 | Adama MA | Oromia | 11, 2007 | 04, 2008 | B | mixed | CS | parasitological | 208 | 14 | 6.7 | 3.73 -  11.04 | CE |
| Giro et al., 2014 | central Oromia | Oromia | 10, 2010 | 05, 2012 | - | - | CS | parasitological | 20059 | 26 | 0.13 | 0.08 - 0.19 | CE |
| Guadu et al., 2012 | Hashim Nur’s Meat Export Abattoir | Oromia | 12, 2010 | 03, 2011 | - | mixed | CS | parasitological | 415 | 164 | 39.5 | 34.78 -  44.40 | *T. hydatigena* |
| Jibat et al., 2008 | HELMEX abattoir | Oromia | 12, 2005 | 06, 2006 | B | mixed | CS | parasitological | 1536 | - | - |  | *T. hydatigena*, CE |
| Kumsa and Mohammedzein, 2012 | Jimma | Oromia | 11, 2010 | 04, 2011 | B | mixed | CS | parasitological | 302 | 75 | 24.8 | 20.06 -  30.11 | CE |
| Mandefro et al., 2015 * | Bishoftu Elfora Export Abattoir | Oromia | 10, 2013 | 04, 2014 | B | mixed | CS | parasitological | 384 | 31 | 8.07 | 5.55 -  11.26 | *T. hydatigena* |
| Mandefro et al., 2015* | Bishoftu Elfora Export Abattoir | Oromia | 10, 2013 | 04, 2014 | B | mixed | CS | parasitological | 384 | 4 | 1.04 | 0.28 - 2.65 | CE |
| Mekuria et al., 2013 | Dire Dawa MA | Dire Dawa | - | - | B | mixed | CS | parasitological | 425 | 112 | 26.4 | 22.20 -  30.70 | *T. hydatigena* |
| Mengistu et al., 2017 | Bishoftu Elfora Export Abattoir | Oromia | 11, 2014 | 04, 2015 | B | mixed | CS | parasitological | 384 | 45 | 11.7 | 8.68 -  15.36 | *T. multiceps* |
| Regassa et al., 2006 | western Oromia | Oromia | 2003 | 2004 | B | mixed | CS | parasitological | 245 | 0 | 0 | 0.00 - 1.49 | Tapeworm infection |
| Regassa et al., 2013 | Luna Export Abattoir | Oromia | 12, 2009 | 04, 2010 | - | - | CS | parasitological | 674 | - | - |  | *T. hydatigena*, *T. ovis*, CE |
| Samuel and Zewde, 2010 | HASHIM-NUR Export Abattoir, Debre Zeit | Oromia | - | - | - | mixed | CS | parasitological | 768 | 358 | 46.6 | 43.04 -  50.21 | *T. hydatigena* |
| Sissay et al., 2008 * | Haramaya, Harar, Dire-Dawa and Jijiga abattoirs | Oro, Har, DD, Som | 05, 2003 | 04, 2005 | B | mixed | CS | parasitological | 632 | 141 | 22 | 19.12 -  25.76 | *T. ovis* |
| Sissay et al., 2008 * | Haramaya, Harar, Dire-Dawa and Jijiga abattoirs | Oro, Har, DD, Som | 05, 2003 | 04, 2005 | B | mixed | CS | parasitological | 632 | 336 | 53 | 49.19 -  57.11 | *T. hydatigena* |
| Sissay et al., 2008 * | Haramaya, Harar, Dire-Dawa and Jijiga abattoirs | Oro, Har, DD, Som | 05, 2003 | 04, 2005 | B | mixed | CS | parasitological | 632 | 412 | 65 | 61.33 -  68.90 | CE |
| Terefe et al., 2019 | Harar, Dire Dawa and Haramaya | Har, DD, Oro | 02, 2015 | 09, 2016 | - | - | CS | para + molecular | 95 | 0 | 0 | 0.00 - 3.81 | CE |
| Teshome et al., 2017 | ELFORA export abattoir | Oromia | 11, 2015 | 04, 2016 | - | mixed | CS | parasitological | 450 | 16 | 3.6 | 2.05 - 5.71 | CE |
| Tigre et al., 2016 | Jimma, AA abattoirs | Oromia | 01, 2010 | 10, 2011 | B | -? | CS | para + molecular | - | 8 | - |  | CE |
| Wondimu et al., 2011 | HNELME abattoir | Oromia | 10, 2010 | 04, 2011 | - | mixed | CS | parasitological | 576 | 368 | 63.9 | 59.82 -  67.82 | *T. hydatigena* |
| Worku, 2017* | Bishoftu Elfora Export Abattoir | Oromia | 11, 2015 | 03, 2016 | B | mixed | CS | parasitological | 400 | 27 | 6.8 | 4.50 - 9.67 | CE |
| Worku, 2017* | Bishoftu Elfora Export Abattoir | Oromia | 11, 2015 | 03, 2016 | B | mixed | CS | parasitological | 400 | 20 | 5 | 3.08 - 7.62 | *T. multiceps* |
