# Supplementary material for: A systematic review and meta-analysis on prevalence and distribution of Taenia and Echinococcus infections in Ethiopia
Source: Parasit Vectors. 2021 Sep 6;14:447. doi: 10.1186/s13071-021-04925-w (PMC8419976; doi:10.1186/s13071-021-04925-w)
Supplement: Supplementary file 7 — Additional file 7: Table S7. Characteristics of studies included in the systematic review and meta-analysis (study subject: camel). F, female; M, male; B = both male and female; CS, cross sectional; p, prevalence; CI, confidence interval. [file 13071_2021_4925_MOESM7_ESM.doc]

| **Reference** | **Study area** | **Region** | **year of study** | | **sex** | **Age (yrs)** | **Study design** | **dx method** | **Sample size** | **no +** | **P (%)** | **95% CI** | **Parasite/ disease category** |
| --- | --- | --- | --- | --- | --- | --- | --- | --- | --- | --- | --- | --- | --- |
| Gebremichael et al., 2013 | Ayssaita District | Afar | 10, 2012 | 05, 2013 | B | mixed | CS | parasitological | 421 | 144 | 34.2 | 29.68 -  38.95 | CE |
| Giro et al., 2014 | central Oromia | Oromia | 10, 2010 | 05, 2012 | - | - | CS | parasitological | 770 | 474 | 61.6 | 58.02 -  65.01 | CE |
| Hailemariam et al., 2012 | Aweday, Jigjiga, haramaya, AA abattoirs | Somali, oromia, AA | 06, 2010 | 02, 2011 | - | - | - | molecular | - | 16 |  |  | CE |
| Terefe et al., 2019 | Harar, Dire Dawa and Haramaya | Har, DD, Oro | 02, 2015 | 09, 2016 | - | - | CS | para + molecular | 25 | 3 | 12 | 2.55 -  31.22 | CE |
| Tigre et al., 2016 | Jimma, AA abattoirs | Oromia | 01, 2010 | 10, 2011 | B | - | CS | para + molecular | - | 32 | - |  | CE |
