# Supplementary material for: A systematic review and meta-analysis on prevalence and distribution of Taenia and Echinococcus infections in Ethiopia
Source: Parasit Vectors. 2021 Sep 6;14:447. doi: 10.1186/s13071-021-04925-w (PMC8419976; doi:10.1186/s13071-021-04925-w)
Supplement: Supplementary file 8 — Additional file 8: Table S8. Characteristics of studies included in the systematic review and meta-analysis (study subject: pig). F, female; M, male; B = both male and female; CS, cross sectional; p, prevalence; CI, confidence interval. [file 13071_2021_4925_MOESM8_ESM.doc]

| **Reference** | **Study area** | **region** | **year of study** | | **sex** | **Age (yrs)** | **Study design** | **dx method** | **Sample size** | **no +** | **P (%)** | **95% CI** | **Parasite/ disease category** |
| --- | --- | --- | --- | --- | --- | --- | --- | --- | --- | --- | --- | --- | --- |
| Ayele et al., 2019 | AA Abattoir Enterprise | Addis Ababa | 01, 2018 | 04, 2018 | B | mixed | CS | parasitological | 251 | 25 | 9.96 | 6.55 -  14.35 | CE |
| Tigre et al., 2016 | Jimma, AA abattoirs | Oromia | 01, 2010 | 10, 2011 | B | - | CS | para + molecular | - | 2 | - |  | CE |
