# Supplementary material for: A systematic review and meta-analysis on prevalence and distribution of Taenia and Echinococcus infections in Ethiopia
Source: Parasit Vectors. 2021 Sep 6;14:447. doi: 10.1186/s13071-021-04925-w (PMC8419976; doi:10.1186/s13071-021-04925-w)
Supplement: Supplementary file 9 — Additional file 9: Table S9. Characteristics of studies included in the systematic review and meta-analysis (study subject: final hosts). F, female; M, male; B = both male and female; CS, cross sectional; p, prevalence; CI, confidence interval. [file 13071_2021_4925_MOESM9_ESM.doc]

| **Reference** | **Study area** | **region** | **year of study** | | **study subject** | **sex** | **Age (yrs)** | **Study design** | **dx method** | **Sample size** | **no +** | **P (%)** | **95% CI** | **Parasite/ disease category** |
| --- | --- | --- | --- | --- | --- | --- | --- | --- | --- | --- | --- | --- | --- | --- |
| Achenef et al., 1999 | Debre Berhan | Amhara | 1996 | 1997 | dog | - | - | CS | parasitological | 17 | 8 | 47.06 | 22.98 -  72.19 | *Taenia* species |
| Degefu and Damet, 2013 | South Wollo | Amhara | 11, 2007 | 02, 2008 | dog | B | - | CS | parasitological | 10 | 2 | 20 | 2.50 -  55.60 | *Echinococcus* spp. |
| Gugsa et al, 2015 * | Mekelle | Tigray | 11, 2009 | 04, 2010 | dogs | B | - | CS | parasitological | 11 | 7 | 63.63 | 30.80 -  89.10 | *T. hydatigina* |
| Gugsa et al, 2015 * | Mekelle | Tigray | 11, 2009 | 04, 2010 | dogs | B | - | CS | parasitological | 11 | 6 | 54.54 | 23.38 -  83.25 | *T. ovis* |
| Gugsa et al, 2015* | Mekelle | Tigray | 11, 2009 | 04, 2010 | dogs | B | - | CS | parasitological | 11 | 6 | 54.54 | 23.38 -  83.25 | *T. multiceps* |
| Jebessa, 2009 | Bale Mountains National Park | Oromia | - | - | wolf | - | - | - | parasitological | 8 | 8 | 100 | 63.10 -  100.00 | *Taenia* species |
| Jones et al., 2012 | Wondo Genet | SNNP | - | - | dog | - | - | CS | parasitological | 62 | 19 | 30 | 19.60 -  43.70 | *E granulosus* |
| Kebede et al., 2009 | Tigray | Tigray | - | - | dog | B | mixed | CS | parasitological | 18 | 3 | 16.7 | 3.60 -  41.40 | *Echinococcus* spp. |
| Kebede, 2019* | Chagni town | Amhara | - | - | dogs | B | mixed | CS | parasitological | 202 | 191 | 94.6 | 90.50 -  97.30 | *Taenia* species |
| Kebede, 2019 * | Chagni town | Amhara | - | - | dogs | B | mixed | CS | parasitological | 9 | 8 | 88.9 | 51.75 -  99.72 | *Echinococcus*  spp. |
| Kebede, 2019 * | Chagni town | Amhara | - | - | dogs | B | mixed | CS | parasitological | 9 | 6 | 66.67 | 29.93 -  92.51 | *T. hydatigina* |
| Kebede, 2019 * | Chagni town | Amhara | - | - | dogs | BB | mixed | CS | parasitological | 9 | 4 | 44.4 | 13.70 -  78.80 | *T. ovis* |
| Koskei et al., 2011 | Asela, Makale, Debrezeit | Oro, Tig | - | - | dog | - | - | - | parasitological | 44 | 15 | 34.1 | 20.50 -  49.90 | *Echinococcus* spp. |
| Mersie, 1993 | Assebe Teferi | Oromia | - | - | dog | B | mixed | CS | parasitological | 9 | 2 | 22 | 2.80 -  60.00 | *Echinococcus* spp. |
| Mulugeta et al., 2019 | Hosanna Town | SNNP | - | - | dog | - | mixed | CS | parasitological | 261 | 11 | 4.2 | 2.10 -  7.40 | *Taenia* species |
| Terefe et al., 2014 | Ethiopia | Eth | 2010 | 2013 | hyena | - | - | - | molecular | 11 | 11 | - |  | *Taenia* species |
| van Kesteren et al., 2015 | Bale Mountains National Park | Oromia | 08, 2008 | 02, 2010 | wolf | - | - | CS | para + molecular | 94 | 4 | 4.3 | 1.20 -  10.50 | Taeniid eggs |
