# Supplementary material for: A systematic review and meta-analysis on prevalence and distribution of Taenia and Echinococcus infections in Ethiopia
Source: Parasit Vectors. 2021 Sep 6;14:447. doi: 10.1186/s13071-021-04925-w (PMC8419976; doi:10.1186/s13071-021-04925-w)
Supplement: Supplementary file 11 — Additional file 11: Table S10. Pooled prevalence of Taenia and Echinococcus infections in intermediate and final hosts by region, Ethiopia. [file 13071_2021_4925_MOESM11_ESM.doc]

| **Characteristics** | **Number of dataset** | **Pooled effect** | | | **Heterogeneity** | | | |
| --- | --- | --- | --- | --- | --- | --- | --- | --- |
|  |  | **Sample size** | **Infected** | **Prevalence (%)** | **95%CI** | **Cochran's Q** | **I^2^ (%)** | **P–value** |
| **CE** | 111 | 96940 | 17971 | 22 | 18-26 | 24420.65 | 100 | 0.000 |
| **Cattle** |  |  |  |  |  |  |  |  |
| Region |  |  |  |  |  |  |  |  |
| Addis Ababa | 3 | 2461 | 647 | 28.4 | 18.5-39.4 | 63.171 | 96.834 | 0.000 |
| Amhara | 18 | 9923 | 1834 | 18.8 | 14.4-23.6 | 597.169 | 97.153 | 0.000 |
| Dire Dawa | 2 | 7977 | 1768 | 21.5 | 19.0-24.1 | 4.995 | 79.979 | 0.025 |
| Harari | 1 | 679 | 77 | 11.3 | 9.05-13.97 | - | - | - |
| Oromia | 23 | 12186 | 4792 | 30.1 | 22.5-38.3 | 1954.511 | 98.874 | 0.000 |
| SNNP | 10 | 4538 | 1299 | 27.1 | 17.1-38.3 | 592.248 | 98.48 | 0.000 |
| Tigray | 7 | 14317 | 3831 | 27.6 | 23.5-31.9 | 166.571 | 96.398 | 0.000 |
| **Sheep** |  |  |  |  |  |  |  |  |
| Region |  |  |  |  |  |  |  |  |
| Addis Ababa | 3 | 1871 | 250 | 8.6 | 0.8-21.8 | 105.943 | 98.112 | 0.000 |
| Amhara | 4 | 1255 | 124 | 7.8 | 3.7-13.3 | 27.707 | 89.172 | 0.000 |
| Dire Dawa | 1 | 166 | 96 | 58 | 49.93-65.44 | - | - | - |
| Harari | 1 | 156 | 116 | 74 | 66.76-81.01 | - | - | - |
| Oromia | 13 | 10804 | 1364 | 19 | 13-25 | 1908.65 | 99 | 0.000 |
| Somali | 1 | 181 | 125 | 69 | 61.78-75.71 | - | - | - |
| Tigray | 1 | 1152 | 134 | 11.6 | 9.84-13.63 | - | - | - |
| **Goat** |  |  |  |  |  |  |  |  |
| Region |  |  |  |  |  |  |  |  |
| Addis Ababa | 3 | 1465 | 127 | 7.0 | 0.00-18.3 | 102.108 | 98.041 | 0.000 |
| Amhara | 1 | 420 | 8 | 1.9 | 0.83-3.72 | - | - | - |
| Dire Dawa | 1 | 159 | 107 | 67 | 59.42-74.52 | - | - | - |
| Harari | 1 | 148 | 93 | 63 | 54.52-70.63 | - | - | - |
| Oromia | 12 | 24479 | 411 | 7.4 | 2.4-14.5 | 1230.386 | 99.106 | 0.000 |
| Somali | 1 | 171 | 118 | 69 | 61.49-75.84 | - | - | - |
| **Camel** |  |  |  |  |  |  |  |  |
| Region |  |  |  |  |  |  |  |  |
| Afar | 1 | 421 | 144 | 34.2 | 29.68-38.95 | - | - | - |
| Oromia | 1 | 770 | 474 | 61.6 | 58.02-65.01 | - | - | - |
| **Pig** |  |  |  |  |  |  |  |  |
| Region |  |  |  |  |  |  |  |  |
| Addis Ababa | 1 | 251 | 25 | 9.96 | 6.55-14.35 | - | - | - |
| **Human** |  |  |  |  |  |  |  |  |
| Region |  |  |  |  |  |  |  |  |
| SNNP | 1 | 990 | 7 | 0.7 | 0.02-1.2 | - | - | - |
| **Echinococcosis** | 7 | 152 | 49 | 33 | 20-48 | 17.24 | 65 | 0.01 |
| **Dog** |  |  |  |  |  |  |  |  |
| Region |  |  |  |  |  |  |  |  |
| Amhara | 2 | 19 | 10 | 54.9 | 0.00-100.0 | 9.309 | 89.258 | 0.002 |
| Oromia | 2 | 38 | 14 | 37.1 | 22.4-53.0 | 0.931 | 0.000 | 0.335 |
| SNNP | 1 | 62 | 19 | 30 | 19.6-43.7 | - | - | - |
| Tigray | 2 | 33 | 6 | 19.0 | 7.2-34.3 | 0.063 | 0.000 | 0.803 |
| **Taeniasis** | 32 | 10504 | 350 | 3 | 2-4 | 279.07 | 89 | 0.000 |
| **Human** |  |  |  |  |  |  |  |  |
| Region |  |  |  |  |  |  |  |  |
| Addis Ababa | 3 | 739 | 35 | 4.0 | 1.1-8.3 | 11.933 | 83.240 | 0.003 |
| Amhara | 6 | 2056 | 73 | 2.3 | 0.3-5.7 | 73.849 | 93.229 | 0.000 |
| Oromia | 5 | 1994 | 80 | 3.9 | 2.4-5.6 | 8.623 | 53.612 | 0.071 |
| SNNP | 10 | 4382 | 124 | 2.5 | 1.5-3.8 | 51.847 | 82.641 | 0.000 |
| Tigray | 1 | 291 | 7 | 2.4 | 0.97-4.89 | - | - | - |
| **Cattle** |  |  |  |  |  |  |  |  |
| Region |  |  |  |  |  |  |  |  |
| Oromia | 1 | 257 | 1 | 0.4 | 0.01-2.15 | - | - | - |
| **Sheep** |  |  |  |  |  |  |  |  |
| Region |  |  |  |  |  |  |  |  |
| Oromia | 2 | 347 | 9 | 3.0 | 0.00-17.5 | 23.472 | 95.740 | 0.000 |
| **Goat** |  |  |  |  |  |  |  |  |
| Region |  |  |  |  |  |  |  |  |
| Oromia | 2 | 336 | 9 | 3.1 | 0.00-17.7 | 23.225 | 95.694 | 0.000 |
| **Wolf** |  |  |  |  |  |  |  |  |
| Region |  |  |  |  |  |  |  |  |
| Oromia | 2 | 102 | 12 | 51.3 | 0.00-100 | 43.568 | 97.705 | 0.000 |
| ***T. saginata (C. bovis)*** | 53 | 111084 | 6435 | 7 | 5-9 | 4458.76 | 99 | 0.000 |
| **Cattle** |  |  |  |  |  |  |  |  |
| Region |  |  |  |  |  |  |  |  |
| Addis Ababa | 4 | 56284 | 1715 | 4.4 | 1.1-9.3 | 738.361 | 99.594 | 0.000 |
| Amhara | 15 | 11570 | 1130 | 4.9 | 2.2-8.5 | 734.307 | 98.093 | 0.000 |
| Dire Dawa | 1 | 6441 | 208 | 3.23 | 2.81-3.69 | - | - | - |
| Harari | 1 | 898 | 177 | 19.7 | 17.16-22.47 | - | - | - |
| Oromia | 22 | 29068 | 2697 | 7.9 | 5.7-10.4 | 811.782 | 97.413 | 0.000 |
| SNNP | 5 | 2139 | 247 | 10.5 | 4.0-19.4 | 131.778 | 96.965 | 0.000 |
| Somali | 1 | 400 | 9 | 2.25 | 1.03-4.23 | - | - | - |
| Tigray | 4 | 4284 | 252 | 6.0 | 4.5-7.8 | 14.545 | 79.375 | 0.002 |
| ***T. hydatigena*** | 30 | 10561 | 3764 | 38 | 29-47 | 2622.37 | 99 | 0.000 |
| **Sheep** |  |  |  |  |  |  |  |  |
| Region |  |  |  |  |  |  |  |  |
| Addis Ababa | 1 | 576 | 45 | 7.81 | 5.75-10.31 | - | - | - |
| Amhara | 1 | 510 | 233 | 45.69 | 41.3-50.12 | - | - | - |
| Dire Dawa | 2 | 586 | 232 | 52.8 | 0.00-100.0 | 189.354 | 99.472 | 0.000 |
| Harari | 1 | 156 | 123 | 79 | 71.59-84.97 | - | - | - |
| Oromia | 9 | 3229 | 1039 | 30.3 | 16.5-46.1 | 656.776 | 98.782 | 0.000 |
| Somali | 1 | 181 | 144 | 80 | 72.94-85.18 | - | - | - |
| **Goat** |  |  |  |  |  |  |  |  |
| Region |  |  |  |  |  |  |  |  |
| Addis Ababa | 1 | 576 | 91 | 15.8 | 12.91-19.04 | - | - | - |
| Amhara | 1 | 420 | 304 | 72.38 | 67.84-76.6 | - | - | - |
| Dire Dawa | 2 | 584 | 204 | 40.7 | 10.5-74.6 | 48.754 | 97.949 | 0.000 |
| Harari | 1 | 148 | 66 | 45 | 36.43-52.98 | - | - | - |
| Oromia | 9 | 3424 | 1185 | 30.2 | 16.4-45.9 | 692.709 | 98.845 | 0.000 |
| Somali | 1 | 171 | 98 | 57 | 49.53-64.83 | - | - | - |
| ***T. ovis*** | 14 | 3753 | 443 | 14 | 9-20 | 328.44 | 96 | 0.000 |
| **Sheep** |  |  |  |  |  |  |  |  |
| Region |  |  |  |  |  |  |  |  |
| Amhara | 1 | 510 | 43 | 8.43 | 6.17-11.19 | - | - | - |
| Dire Dawa | 1 | 166 | 22 | 13 | 8.5-19.38 | - | - | - |
| Harari | 1 | 156 | 45 | 29 | 21.88-36.63 | - | - | - |
| Oromia | 3 | 920 | 72 | 8.3 | 0.6-2.15 | 57.282 | 96.508 | 0.000 |
| Somali | 1 | 181 | 62 | 34 | 27.37-41.66 | - | - | - |
| **Goat** |  |  |  |  |  |  |  |  |
| Region |  |  |  |  |  |  |  |  |
| Amhara | 1 | 420 | 36 | 8.57 | 6.08-11.62 | - | - | - |
| Dire Dawa | 1 | 159 | 37 | 23 | 16.94-30.62 | - | - | - |
| Harari | 1 | 148 | 33 | 22 | 15.87-29.86 | - | - | - |
| Oromia | 3 | 922 | 48 | 5.4 | 0.5-14.0 | 34.864 | 94.263 | 0.000 |
| Somali | 1 | 171 | 45 | 26 | 19.89-33.58 | - | - | - |
| ***T. multiceps*** | 7 | 2541 | 151 | 5 | 2-10 | 110.26 | 95 | 0.000 |
| **Sheep** |  |  |  |  |  |  |  |  |
| Region |  |  |  |  |  |  |  |  |
| Amhara | 1 | 220 | 42 | 19.09 | 14.12-24.92 | - | - | - |
| Oromia | 3 | 1153 | 42 | 3.6 | 2.4-5.1 | 2.962 | 32.471 | 0.001 |
| **Goat** |  |  |  |  |  |  |  |  |
| Region |  |  |  |  |  |  |  |  |
| Oromia | 3 | 1168 | 67 | 4.8 | 0.00-12.4 | 56.501 | 96.460 | 0.000 |
