# Supplementary figures and images for: A systematic review and meta-analysis on prevalence and distribution of Taenia and Echinococcus infections in Ethiopia
Source: Parasit Vectors. 2021 Sep 6;14:447. doi: 10.1186/s13071-021-04925-w (PMC8419976; doi:10.1186/s13071-021-04925-w)

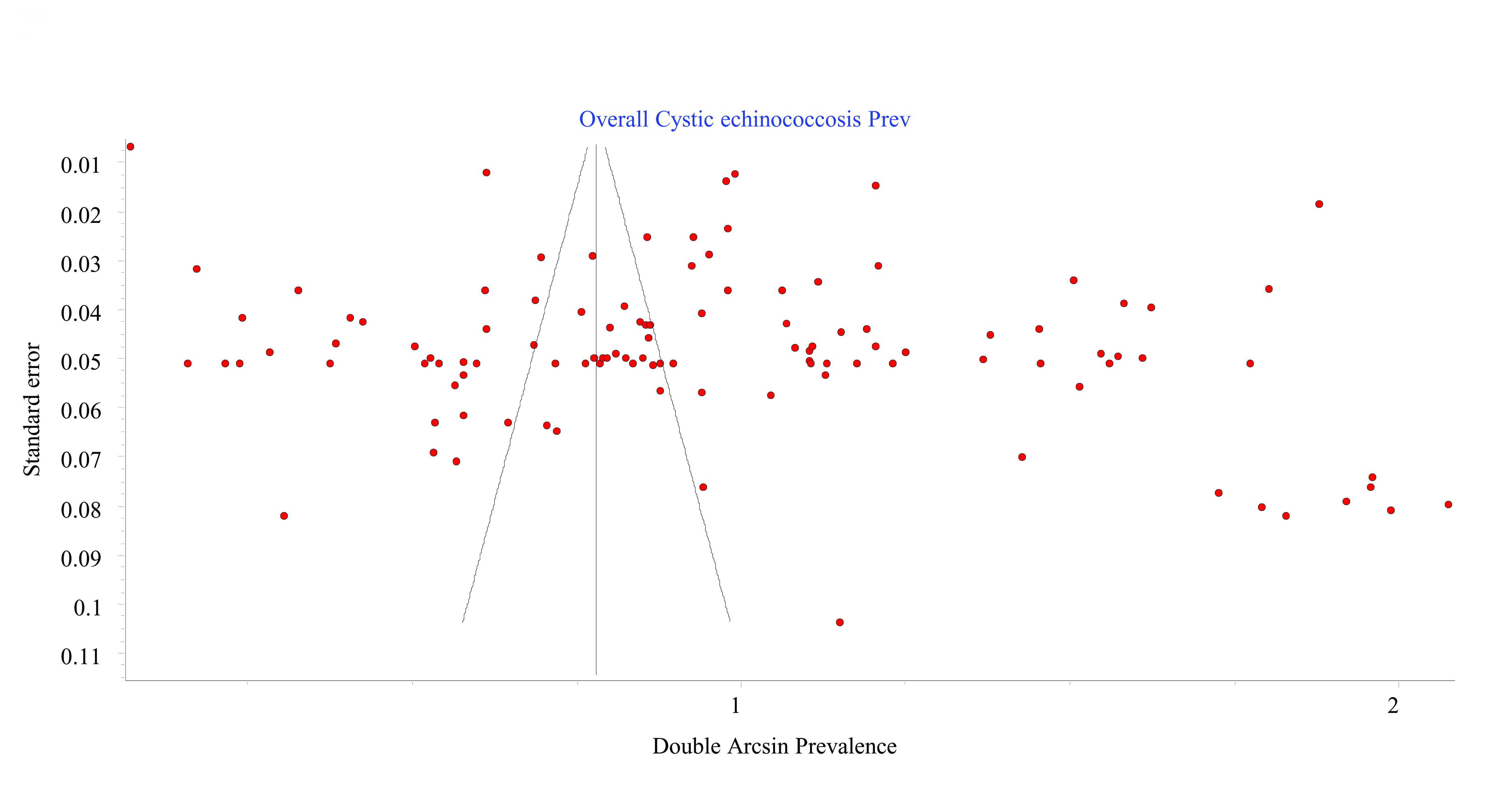

Supplement: Supplementary file 12 — Additional file 12: Figure S2. Publication bias evidenced by funnel plots for overall prevalence of cystic echinococcosis. Prev, prevalence. [file 13071_2021_4925_MOESM12_ESM.tif]

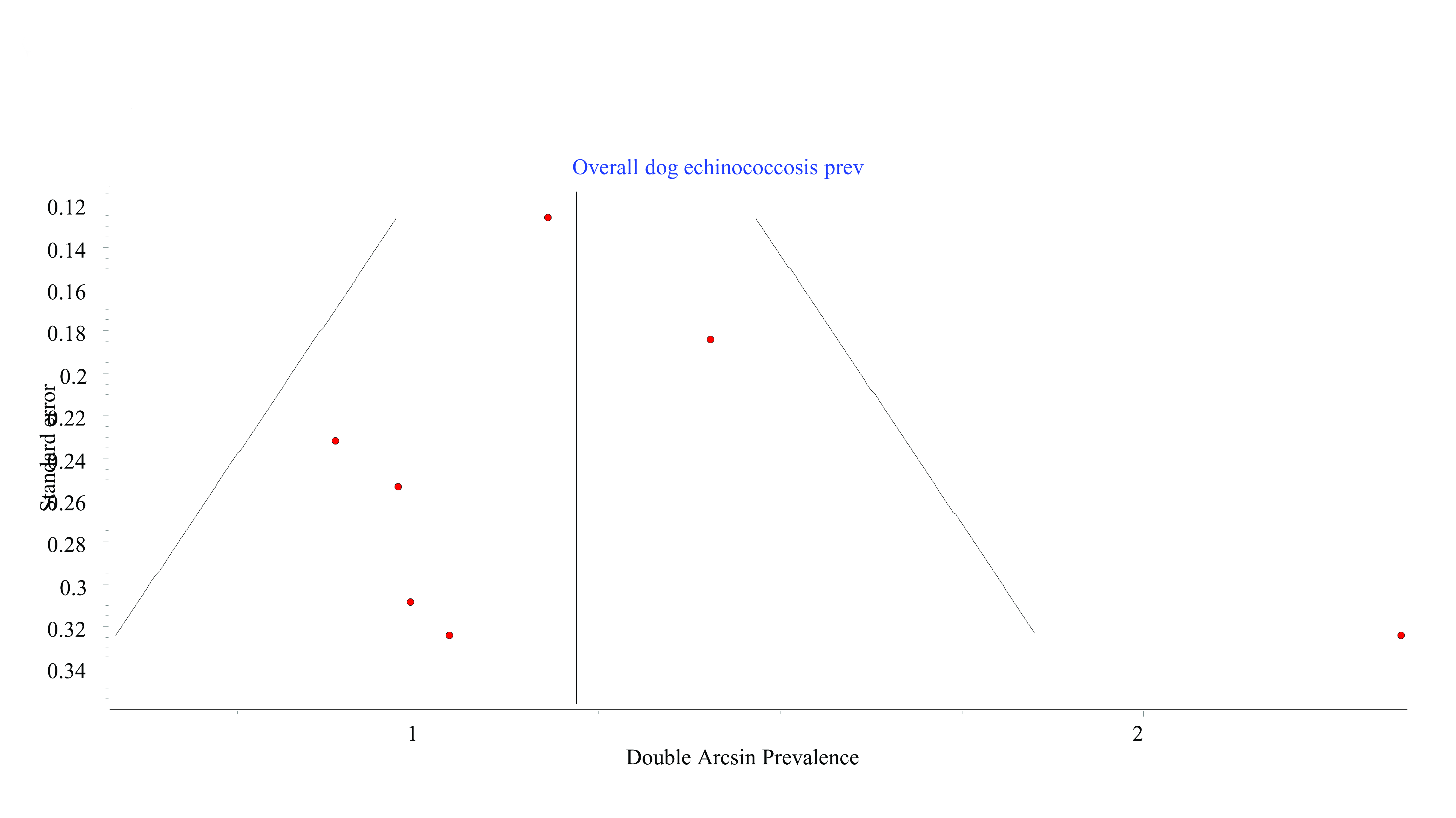

Supplement: Supplementary file 13 — Additional file 13: Figure S3. Publication bias evidenced by funnel plots for overall prevalence of dog echinococcosis. Prev, prevalence. [file 13071_2021_4925_MOESM13_ESM.tif]

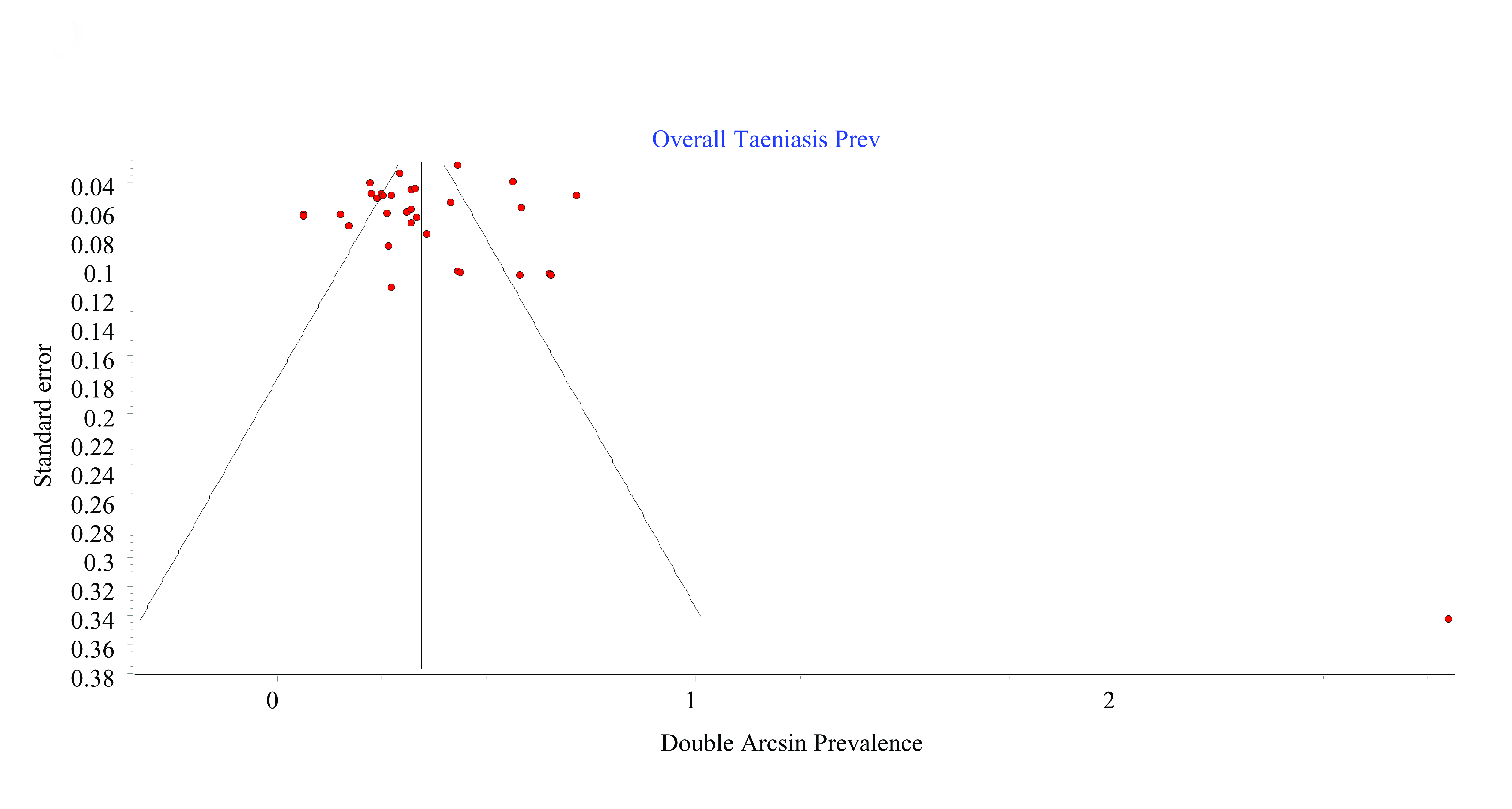

Supplement: Supplementary file 14 — Additional file 14: Figure S4. Publication bias evidenced by funnel plots for overall prevalence of taeniasis. Prev, prevalence. [file 13071_2021_4925_MOESM14_ESM.tif]

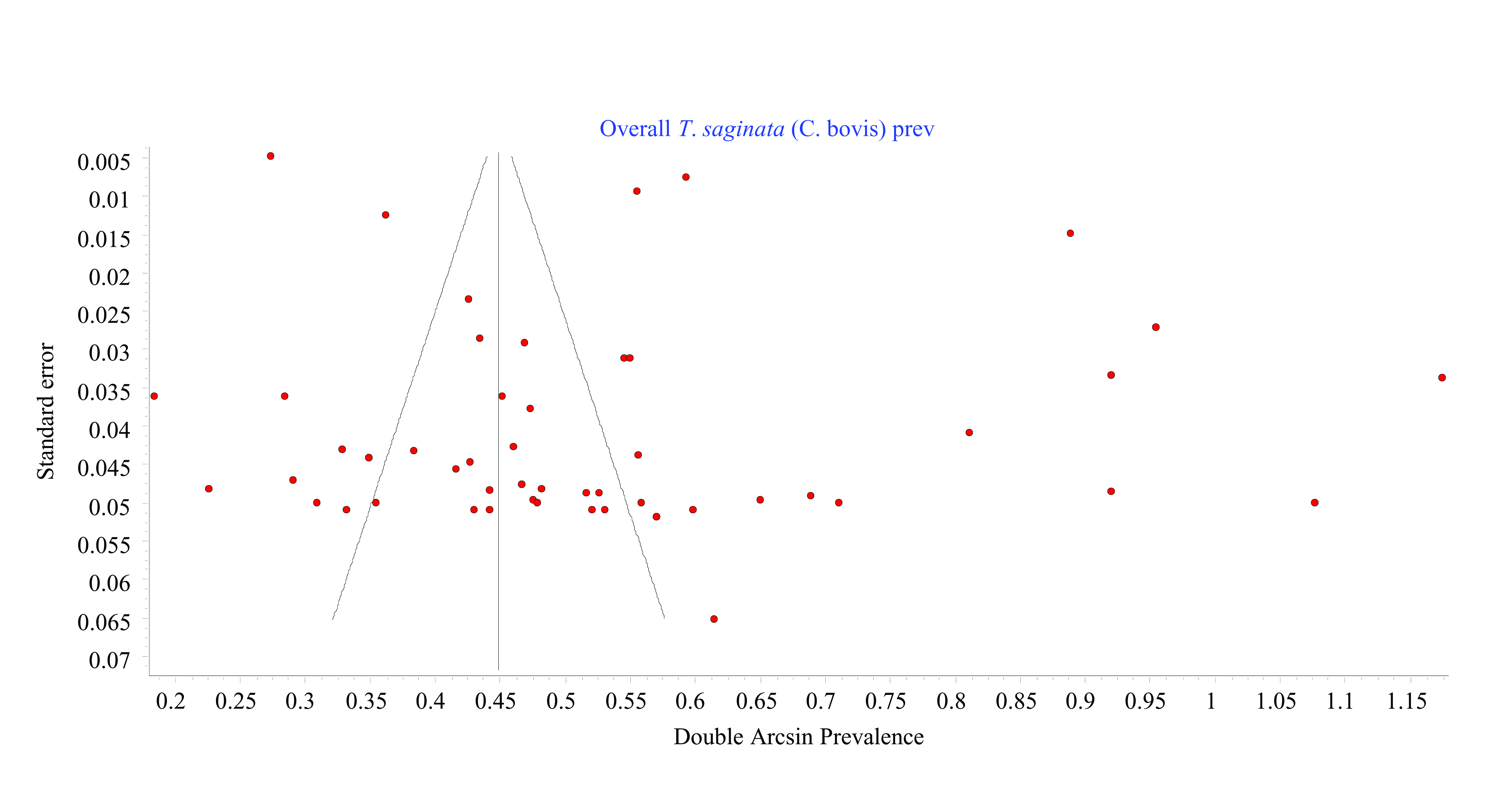

Supplement: Supplementary file 15 — Additional file 15: Figure S5. Publication bias evidenced by funnel plots for overall prevalence of T.saginata (C. bovis). Prev, prevalence. [file 13071_2021_4925_MOESM15_ESM.tif]

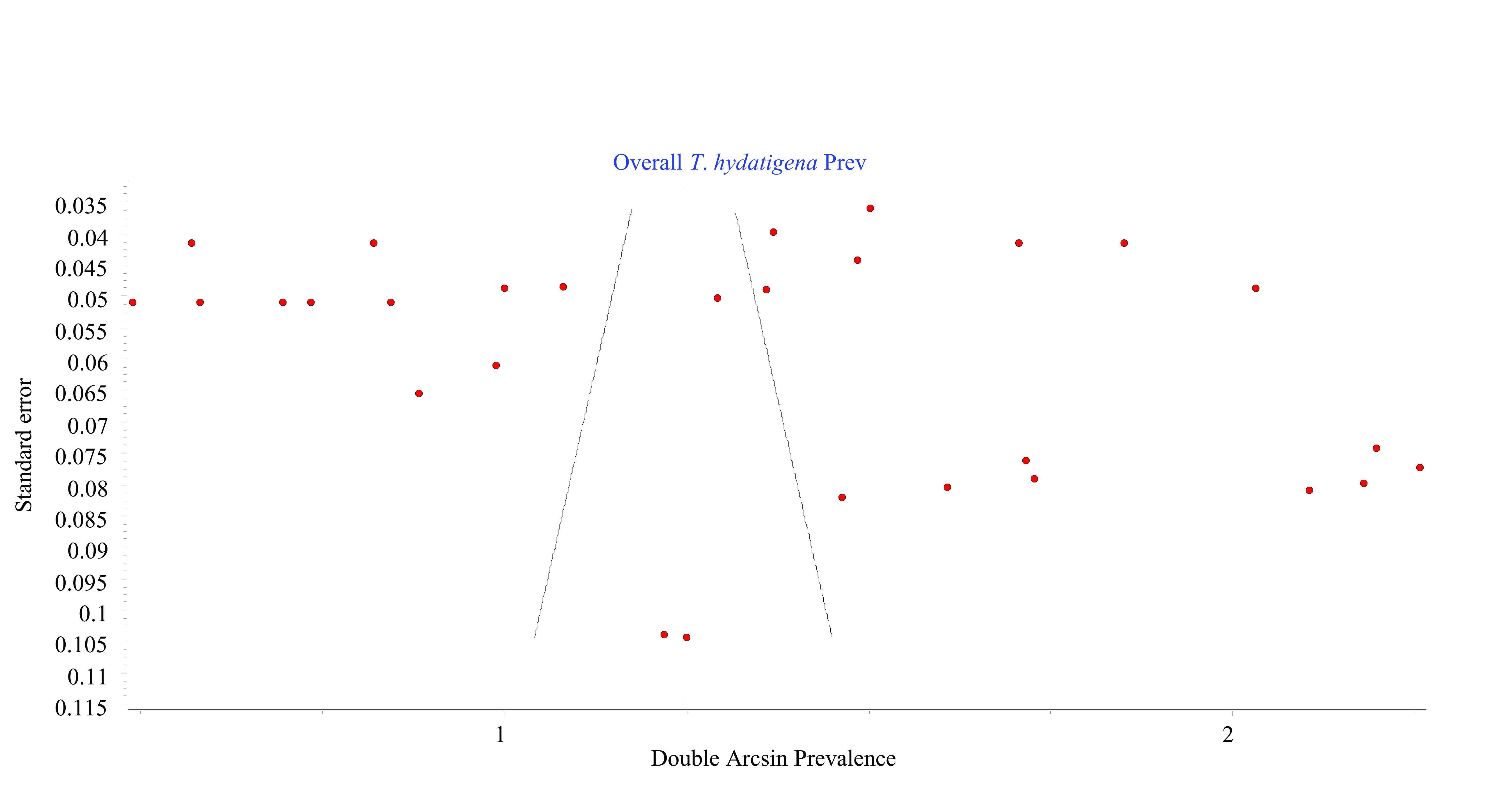

Supplement: Supplementary file 16 — Additional file 16: Figure S6. Publication bias evidenced by funnel plots for overall prevalence of T. hydatigena. Prev, prevalence. [file 13071_2021_4925_MOESM16_ESM.tif]

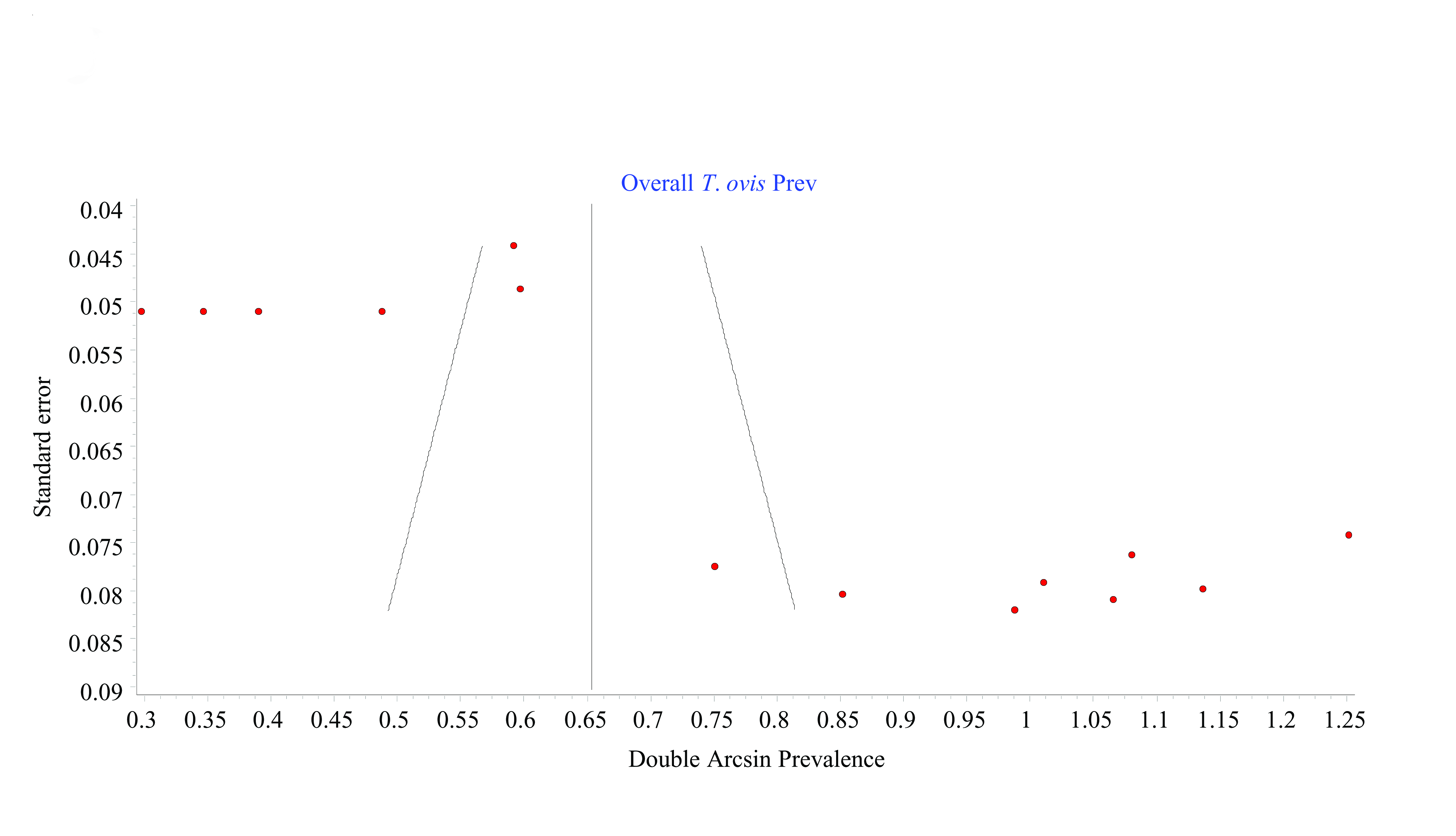

Supplement: Supplementary file 17 — Additional file 17: Figure S7. Publication bias evidenced by funnel plots for overall prevalence of T. ovis. Prev, prevalence. [file 13071_2021_4925_MOESM17_ESM.tif]

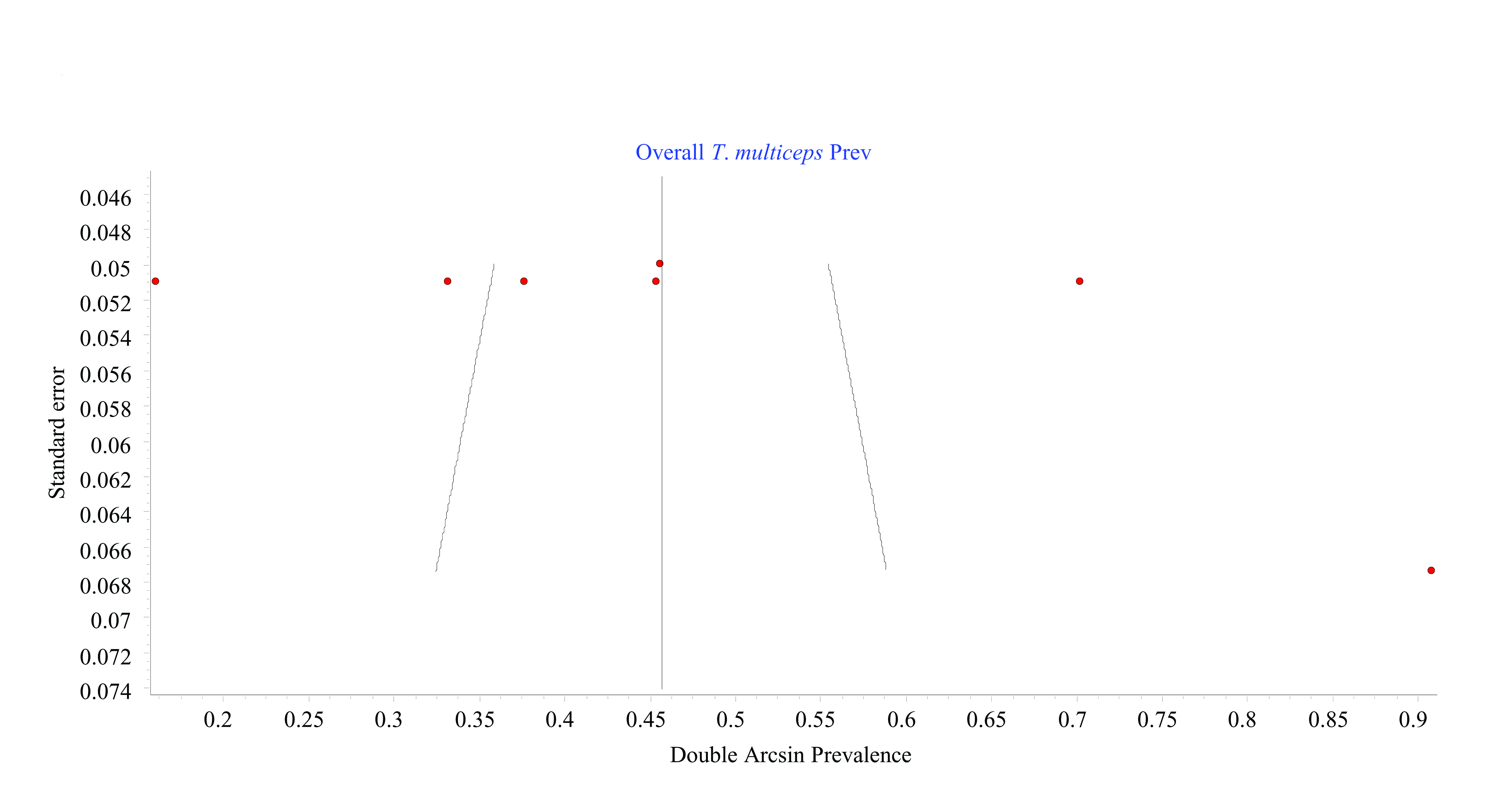

Supplement: Supplementary file 18 — Additional file 18: Figure S8. Publication bias evidenced by funnels plots for overall prevalence of T. multiceps. Prev, prevalence. [file 13071_2021_4925_MOESM18_ESM.tif]
